# Knowledge‐based radiation treatment planning: A data‐driven method survey

**DOI:** 10.1002/acm2.13337

**Published:** 2021-07-07

**Authors:** Shadab Momin, Yabo Fu, Yang Lei, Justin Roper, Jeffrey D. Bradley, Walter J. Curran, Tian Liu, Xiaofeng Yang

**Affiliations:** ^1^ Department of Radiation Oncology and Winship Cancer Institute Emory University Atlanta GA USA

**Keywords:** data‐driven methods, deep learning, knowledge‐based planning, machine learning, radiation dose prediction methods, radiotherapy treatment planning

## Abstract

This paper surveys the data‐driven dose prediction methods investigated for knowledge‐based planning (KBP) in the last decade. These methods were classified into two major categories—traditional KBP methods and deep‐learning (DL) methods—according to their techniques of utilizing previous knowledge. Traditional KBP methods include studies that require geometric or anatomical features to either find the best‐matched case(s) from a repository of prior treatment plans or to build dose prediction models. DL methods include studies that train neural networks to make dose predictions. A comprehensive review of each category is presented, highlighting key features, methods, and their advancements over the years. We separated the cited works according to the framework and cancer site in each category. Finally, we briefly discuss the performance of both traditional KBP methods and DL methods, then discuss future trends of both data‐driven KBP methods to dose prediction.

## 1 INTRODUCTION

Cancer is the second‐leading cause of death in North America with the most common types being the cancer of lung, breast, and prostate.^1^ Radiation therapy (RT), chemotherapy, surgery or their combination are used to control the disease. Approximately 50% of all cancer patients undergo RT during the course of their illness,^2^ which makes RT a crucial component of cancer treatments. Technological innovations have driven the transition from conformal RT to intensity‐modulated radiation therapy (IMRT) resulting in significant improvements in the twofold dosimetric goal of preferentially sparing critical organs‐at‐risk (OARs) while improving high dose conformity to the target. Furthermore, algorithmic advancements have also played major roles in enhancing the efficiency of RT treatments. These include the transition from forward treatment planning to inverse treatment planning approaches and extension of static field IMRT to volumetric‐modulated arc therapy (VMAT). However, despite the use of complex inverse optimization algorithms, an inverse planning approach typically demands a large amount of manual effort and considerable skills to generate a high‐quality treatment plan with the desired dose distribution. This process often spans several days between patient simulation and when RT begins.

To improve the treatment planning efficiency, data‐driven treatment planning approaches have been investigated that use knowledge from prior cases to predict the outcome of a new case. For example, this concept was utilized by researchers over a decade ago in the form of knowledge‐based planning (KBP) to predict dose in radiotherapy treatment planning.^3^ In this review, we group data‐driven KBP methods into two major categories: I) Traditional KBP methods II) Deep learning (DL)‐based KBP methods. Traditional KBP includes methods that utilize various anatomical and geometrical features (distance to target structures, volumes of target, and OAR structures, etc.) to build a mathematical or statistical model that is then used to predict various dosimetry features (i.e., dose–volume metrics, dose–volume histogram (DVH), spatial dose distribution, etc.) for a new case.^4^ Traditional KBP methods have been widely investigated in the last decade and have also been clinically implemented. The commercial software RapidPlan^TM^ is one example of the KBP module based on a traditional method, which was released in 2014 by Varian Medical Systems (Varian Medical Systems). The traditional KBP methods include atlas‐based, statistical modeling, and machine learning (ML) methods. In general, traditional KBP methods utilize geometric features (i.e., OAR distance to the planning target volume (PTV) and OAR overlap volume histogram (OVH)) either to find the best‐matched prior case(s) from a repository or to build dose prediction models (i.e., ML, statistical model). In this review, ML‐based methods are included in the traditional KBP category as ML follows a similar framework to traditional KBP methods in terms of inputs (handcrafted geometric and anatomical features) and outputs (DVH metrics).

Prior to the investigations of DL for the dose prediction task, DL had been extensively studied for various imaging tasks including image registration, segmentation, etc., in which DL methods have gained considerable momentum, outperforming many of the existing state‐of‐art techniques.^5^, ^6^, ^7^, ^8^, ^9^, ^10^, ^11^, ^12^, ^13^, ^14^, ^15^, ^16^, ^17^, ^18^, ^19^, ^20^, ^21^ The enhanced performance of DL in imaging and vision tasks can be attributed to the design of a convolutional neural network (CNN), a class of deep neural networks (DNN) with regularized multilayer perceptron.^22^ In the past few years, researchers have investigated various DL network architectures for KBP. As an example, U‐Net,^23^ originally designed for image segmentation, has recently been used to predict the radiation dose distribution without the need for the complex dose calculations routinely used for treatment planning.^24^, ^25^, ^26^, ^27^ In contrast to traditional KBP methods that use handcrafted features, DL methods can automatically learn image features that are tailored to the specific prediction task from the raw data (i.e., CT, contour, dose map, etc.) Therefore, a key difference between traditional and DL‐based KBP is the way in which previous knowledge is utilized.

KBP research is expanding exponentially, and while there are excellent review papers in publication, this current review is needed to address recent advancements in dose prediction methods. Previous review papers are limited to only traditional KBP methods,^28^, ^29^ multicriteria optimization methods,^30^ and multicriteria optimization as well as traditional KBP methods^31^ for automatic treatment planning. Another review provided a semi‐comprehensive review of 62 publications including both machine learning and DL‐based methods for automatic treatment planning; however, this study is not specific to the dose prediction task and also includes articles on other delivery parameters (i.e., beam orientation, arc lengths, etc.).^32^ In comparison to previous review papers, this work focuses solely on the dose prediction task and presents a comprehensive review by separating over 120 publications on data‐driven KBP methods published by August 2020 into two categories: the traditional KBP methods and the recently emerging DL‐based methods. For each category, we first present a review of key features and methods (i.e., OVH, distance to target histogram (DTH) in traditional KBP methods, and different neural networks in DL‐based methods). Subsequently, we present the literature on outcomes of KBP methods according to the influence of various clinically applicable parameters (i.e., outliers, data inconsistency, sample size, treatment planning efficiency and site, multi‐modality, and multi‐institutional investigations). Finally, we discuss the advantages and challenges of each KBP method, followed by an assessment of potential future trends in data‐driven dose prediction methods. Therefore, this current review differentiates itself from previous review papers by the large number of articles covered including more recent publications, the summary of key features, and methodological differences between the published methods, and the focus on the dose prediction task.

## 2 METHODS

### 2.1 Article search and selection

We queried papers from Elsevier Scopus®, Web of Science, PubMed, Google Scholar, and medical physics category of arXiv.org using logical statements that included the following keywords: knowledge‐based treatment planning, machine learning, deep learning, dose prediction, RapidPlan^®^, treatment planning automation, artificial neural network, convolutional neural network, and generative adversarial network. Only peer‐reviewed KBP research articles were included in this review. Each research article from the literature search was manually sorted based on the information presented in the abstract, which was followed by a further in‐depth review of the article. This review focuses on external beam therapies (e.g., IMRT, VMAT, Tomotherapy, Proton), while manuscripts on brachytherapy and patient‐specific quality assurance were excluded. Articles on dose prediction tasks were selected for this review. *Dose prediction* is a broad term that includes the prediction of the entire DVH curve, specific dose metrics (e.g., dose–volume value, mean, max dose), voxel dose, spatial dose distribution (either 2D or 3D), objective weights/constraints based on previous knowledge and the transfer of these metrics to a new case for planning.

## 3 RESULTS

**FIGURE 1:**
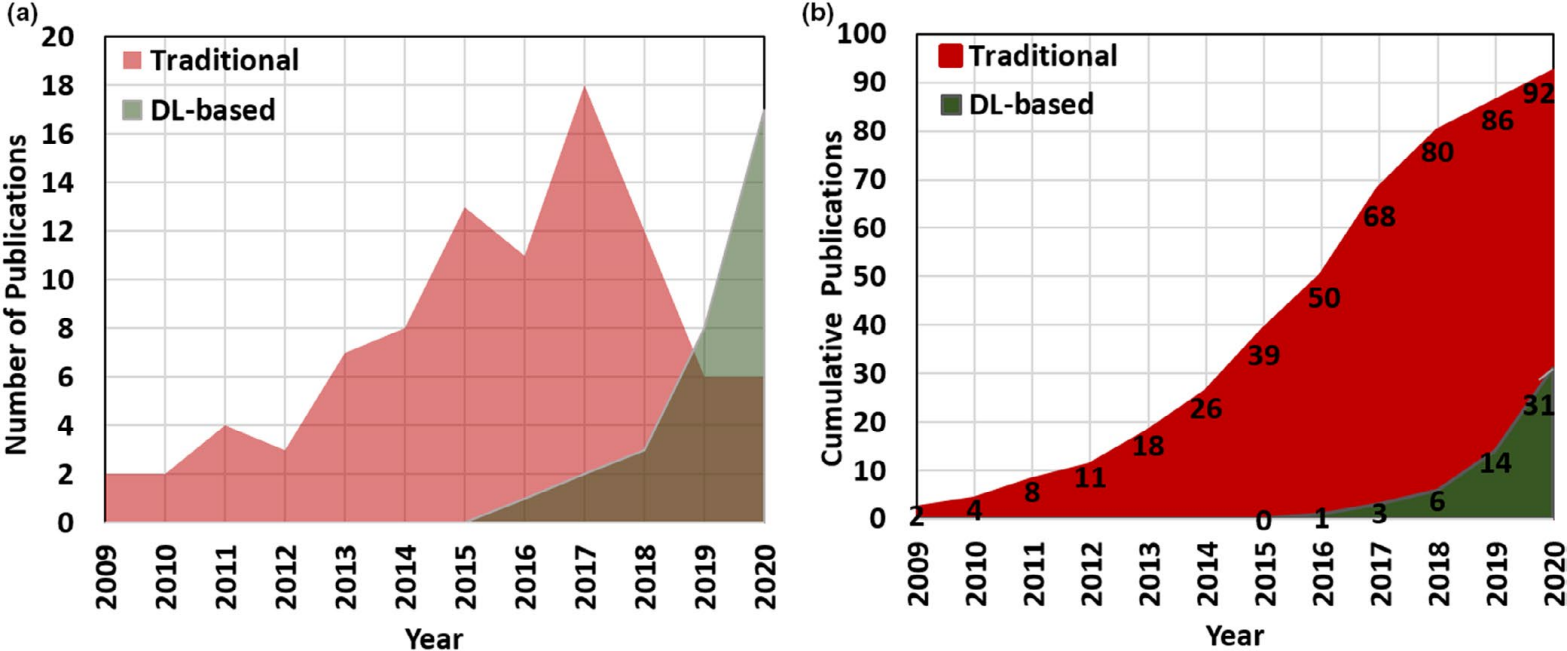
The number of dose prediction publications per year (a) and cumulative number of publications (b) on traditional and DL‐based KBP methods

Figure 1 shows the number of publications per year (Figure 1a) as well as cumulative publications (Figure 1b) for both traditional KBP and DL‐based dose predictions until August 2020. Between 2009 and 2014, there was a gradual increase in the number of publications (Figure 1b) in what appears to be the initial development stage of traditional KBP‐based methods. The cumulative publication curve (Figure 1b) demonstrates an increased rate in the number of traditional KBP‐based articles between 2015 and 2018. Between 2018 and 2020, most studies on traditional KBP methods have been based on a commercial KBP module rather than further development of earlier ML or statistical methods.^33^, ^34^, ^35^, ^36^, ^37^, ^38^, ^39^, ^40^, ^41^, ^42^, ^43^, ^44^, ^45^ This is certainly not because traditional KBP methods have been fully explored, but presumably due to the wider availability of a commercial KBP module in clinical practice and increased interest in exploring DL methods owing to their recent success in achieving state‐of‐the‐art performance in many medical applications. In recent years, a wide array of research on developing new prediction models has transitioned from traditional KBP methods to DL KBP methods.

In the past few years, the number of publications on DL‐based image processing has increased exponentially. More recently, researchers have applied DL methods to dose prediction tasks. In the last 4 years, the number of publications on DL‐based dose predictions has increased from 1 in 2016 to 17 as of August 2020 as shown in Figure 1a. This recent trend shows an increased rate of publications on DL‐based versus traditional KBP methods.

### 3.1 *Knowledge*‐*based planning*

This section includes a review of over 92 articles only on traditional KBP‐based dose prediction methods. We separate these articles based on three dose prediction metrics: I) prediction of the entire DVH (Table 1), II) prediction of one or more dose–volume metrics (Table 2), and III) the voxel‐based dose prediction (Table 3). The articles listed in Table 1 aim to predict the entire DVH for a new patient case and utilize the predicted DVHs to guide the treatment planning. Commercially available software (i.e., RapidPlan^TM^) estimates DVHs and generates optimization objectives for a new plan as shown in Table 1. Table 2 summarizes the articles that predict one or more dose metrics used to guide treatment planning for a new case. Table 3 lists the publications that predict the voxel‐level dose distributions to either assist in plan optimization or automatically generate a new plan for treatment delivery.

**TABLE 1:** Traditional KBP studies aimed to predict dose–volume histograms (DVHs) for providing a starting point for the plan optimization process

Ref.	Method	Approach/Model	Key features	Purpose
46	MB	Support Vector Regression	Organ volumes, shape and DTH	To model functional relationship between DVH and patient anatomical shape information.
4	MB	Fitting using least square min.	OAR distance to PTV	To translate key feature correlation to mathematical relationships between OAR geometry and expected dose.
47	MB	Stepwise multiple regression	DTH	To build feature models to identify the variation of anatomical features contributing to OAR dose sparing.
48	MB	Stepwise multiple regression	Target, OARs, overlap volumes and DTH	Extension of Yuan et al. for intra‐treatment‐modality model (IMRT – Tomotherapy)
49	MB	Stepwise multiple regression	Target, OARs, overlap volumes and DTH, fraction of OAR outside treatment field	To build two predictive models (single‐sparing and standard model) to characterize the dependence of parotid dose sparing on patient anatomical features in the summed (primary + boost) plan, rather than two completely separate models.
50	AB	Direct	Overlapping volume	To select a reference plan from a library of clinically approved/delivered plans with similar medical conditions and geometry
51	AB	Direct	PTV shape, volume, three spherical coordinates of PTV with respect to OAR OVH	To develop a knowledge‐driven decision support system to assist clinicians to pick plan parameters and assess radiation dose distribution for a perspective patient
52	MB	Kernel Density Estimate	Distance to PTV	To develop an automated treatment planning solution that iteratively optimize training set predicts DVHs for OARs generates clinically acceptable plans
53	MB	Ensemble	Anatomical features, DTH	To combine strengths of various linear regression models to build a more robust model
54	MB	K‐nearest neighbors	Generalized‐DTH	To characterize DVH variance in multiple target plans
33, 34, 35, 36, 37, 38, 39, 40, 42, 55, 56, 57, 58, 59, 60, 61, 62, 63, 64, 65, 66, 67, 68, 69, 70, 71, 72, 73, 74, 75, 76, 77, 78				RapidPlan^TM^ Eclipse^®^ treatment planning software: Algorithm is divided into two components: 1) Model configuration and 2) DVH estimation. Mode configuration is divided into data extraction phase and model training phase DVH estimation consists of DVH estimation phase and objective generation phase

Abbreviations: AB, atlas‐based (Direct or Indirect method); DTH, distance‐to‐target histogram; MB, model‐based; OVH, overlap volume histogram.

**TABLE 2:** Traditional KBP studies aimed to predict one or more dose metrics for providing a starting point for the plan optimization process

Ref.	Method	Approach/Model	Key features	Purpose
79	MB	Support vector regression	OAR, DV constraint settings	To create an accurate IMRT plan surface as a decision support tool to aid treatment planners
80	AB	Direct	Clinical stage, and Gleason score	To update the weights of the difference clinical parameters for a new patient through a group‐based simulated annealing approach
81	AB	Direct	OVH	To use geometric and dosimetric information retrieved from a database of previous plans to predict clinically achievable dose–volume metric (*A retrospective based on a method by* ^3^)
82	AB	Direct	OVH	To implement the OVH‐based automated planning system to improve quality, efficiency, and consistency for head and neck cancer
83	AB	Direct	OVH	To predict dose to 35% of rectal volume as a treatment planning quality assurance for prostate cancer patients.
84	AB	Direct	OVH	To investigate if OVH‐driven IMRT database can guide and automate VMAT planning for head and neck cancer
85	MB	Linear Regression	OVH	To evaluate OVH metric for the prediction of rectal dose following hydrogel injection
86	MB	Stepwise multiple regression	OVH	To utilize patients’ anatomic and dosimetric features to predict the pareto front.
87	MB	Logistic Regression	Distance to the tangent field edge	To predict left anterior descending artery maximum dose. Model to guide the positioning of the tangent field to keep maximum dose <10 Gy
88	MB	Linear Regression	OAR volumes	To develop a model to predict attainable prescription dose for IMRT of entire hemithoracic pleura
89	MB	Curve Fitting	Rectum‐target overlap	To predict optimum average rectum dose
90	MB	Stepwise Regression	Target OAR overlap	To predict mean parotid dose
91	AB	Direct	OVH’, In field OAR volumes	The minimum DVH value at the percentage volume of the bladder and rectum was used

Abbreviations: AB, atlas‐based (Direct or Indirect method); DTH, distance‐to‐target histogram; MB, model‐based; OVH, overlap volume histogram.

**TABLE 3:** Traditional KBP studies aimed to predict voxel‐level doses for providing a starting point for the plan optimization process

Ref.	Method	Approach/Model	Key features	Purpose
92	AB	Direct	BEV projections	To identify similar patient cases by matching 2D BEV projections of structures
93	AB	Direct	BEV projections	To adapt the best match plan parameters from one institute to optimize the query case of an outside institution
94, 95	MB	Multivariate analysis Slice weight function	Distance‐to‐PTV, Slice level	To determine the relationship between the position of voxels and corresponding doses to predict the sparing of OARs
96	MB	Active shape model, active optical flow model	PTV locations in relation to spinal cord	To study the effect of PTV contours on dose distribution at the spinal cord. Five subgroups were created according to the PTV locations in relation to spinal cords.
97	AB	Direct	Target‐OAR overlap Shell creation surrounding the match target volume	To adapt the matched case from the database for query case by deforming the match beam fluences, warping the matched primary/boost dose distribution, and distance scaling factor
98	AB	Direct	Target‐OAR overlap	To transfer the beam settings and multileaf collimator positions of the best match case to the new case
99	AB	Direct	The PTV and Seminal vesicles (SV) concaveness angle and % distance from SV to the PTV	To transfer treatment parameters of the atlas case to the new case
100	AB	Indirect	Multi‐scale image appearance features	To use contextual atlas regression forest (cARF) augmented with density estimation over the most informative features to learn an automatic atlas‐selection metric for dose prediction
101	AB	Indirect	Features based on the spatial dose distribution and features derived from DVHs	To extend CRF by introducing the conditional random field model (cARF‐CRF) to transform the probabilistic dose distribution into a scalar dose distribution that adheres to desired DVHs.
102	AB	Indirect	Multi‐scale image appearance features	To converts a predicted per voxel dose distribution into a complete radiotherapy plan through a fully automated pipeline using cARF‐CRF.

Abbreviations: AB, atlas‐based (Direct or Indirect method); DTH, distance‐to‐target histogram; MB, model‐based; OVH, overlap volume histogram.

Atlas‐based and model‐based methods are the two most common traditional KBP techniques for dose prediction (Tables 1, 2, 3). Briefly, in atlas‐based approaches, physical features (e.g., OVH, beam's eye view projections, tumor location) are first identified to determine the similarity between prior clinical plans and a new patient plan. This is followed by transfer of knowledge (i.e., dose constraints, DVH values, beam geometrical parameters, DVHs of best‐matched cases) to predict achievable DVHs of a new case or to provide a better starting point to a treatment planner for further trial‐and‐error optimization. Within atlas‐based methods, an indirect approach selects matching cases based on the dosimetric parameters predicted through models. In contrast, a direct approach compares the similarity between the old and new patient based on features of the plan (i.e., DVHs), CT images or beam's eye view (BEV) projections of structures, and adopts planning parameters of the best match matching cases. In model‐based approaches, statistical or ML models are trained using prior treatment plans. These methods require careful design and selection of handcrafted features such as PTV‐OAR overlap volume, OVH values, OAR distance‐to‐PTV to predict DVH, one or more dose–volume metric or voxel‐level dose distribution by different regression models as summarized in Tables 1, 2, 3.

**FIGURE 2:**
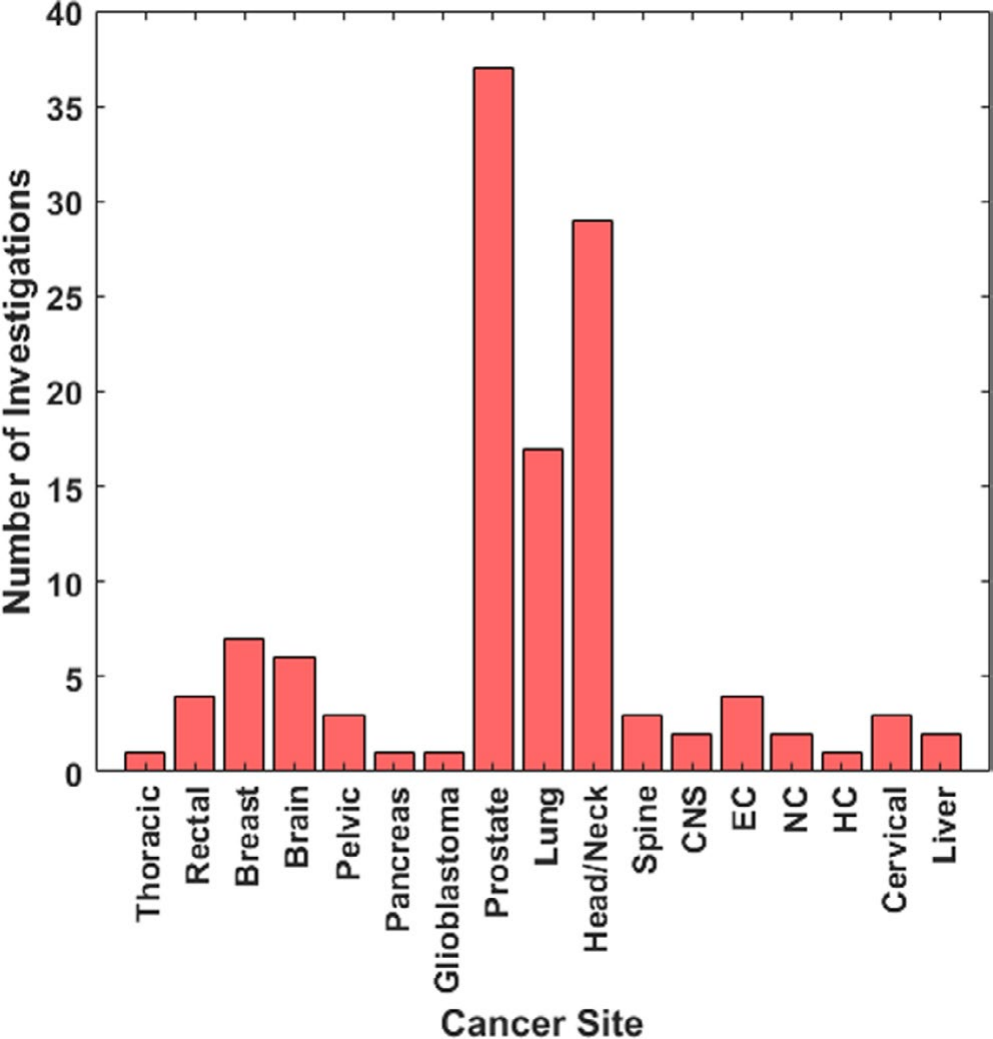
The total number of traditional KBP investigations on dose prediction for various cancer sites. EC, Esophageal cancer; NC, Nasopharyngeal carcinoma; HC, Hepatocellular Cancer

Figure 2 demonstrates the total number of investigations on traditional KBP methods for various treatment sites. Prostate, head and neck, and lung cancers were among the most frequently investigated disease sites, whereas very few studies investigated disease sites in the abdomen, brain, and or the more challenging hemi‐thoracic pleural anatomy.

In Section 3.1.1, 3.2.3, we briefly discuss a key concept of traditional KBP methods. Since different geometric and anatomical features play a major role in traditional KBP‐based methods (Tables 1, 2, 3), we present a review of the most common features/metrics and their advancements over the years in Section 3.1.2, 3.2.3. In Section 3.1.3, 3.2.3, we present literature on the outcomes of KBP methods according to the influence of various clinically relevant parameters (i.e., outliers, data inconsistency, sample size, treatment planning efficiency and site, multi‐modality, and multi‐institutional reports).

#### 3.1.1 Dimensionality reduction

The high dimensional input feature space can be redundant, irrelevant, and correlated, which may result in inefficient training, model overfitting, reduced accuracy, and reduced generalizability of the model. Therefore, it is important to utilize the most discriminative features, rather than including all possible features. To address this during the implementation of a KBP‐based dose prediction model, dimensionality reduction methods are often used in KBP studies to decrease the number of variables through feature extraction and feature selection process. Feature extraction begins with an initial set of variables followed by redefinition that makes them more informative. Principal component analysis (PCA) is a widely used technique for reduced‐order modeling. PCA determines the features that vary the most among the data for representation in a smaller number of dimensions.^103^ For example, in a binary classification problem, the goal is to classify an object A, represented by P number of features in a P‐dimensional vectors. If P is very large, then some characteristics are likely more valuable than others for the purpose of classification. The goal of PCA is to reduce the dimensionality of the original correlated dataset into a smaller set of uncorrelated variables.^103^ The subsequent process of feature selection involves choosing features that are most important for the dose prediction task. In many traditional KBP studies,^46^, ^47^, ^48^, ^49^, ^53^, ^55^, ^56^, ^57^, ^58^, ^59^, ^60^, ^61^, ^62^, ^63^, ^64^, ^65^, ^66^, ^67^, ^68^, ^86^, ^104^, ^105^, ^106^ PCA is commonly used in the process of feature selection.

#### 3.1.2 *Various features*/*metrics*

A common theme in the majority of traditional KBP methods is that the geometric relationship of the target with respect to nearby critical structures correlates with achievable plan quality metrics. Various geometric features have been correlated with dose. Commonly reported geometric features include OVH, distance to target histograms (DTH), and OAR distance‐to‐PTV. As an example, the influence of parotid size and proximity to the PTV was first studied by Hunt et al. and found to be predictive of the achievable parotid sparing.^107^ In addition to 3D geometric features, traditional KBP methods have also incorporated plan features such as mutual information through the beam's eye view projections, and even the number, energy, and angle of the radiation beams (Tables 1, 2, 3). A list of the key features used in traditional KBP studies is tabulated in Tables 1, 2, 3 along with their corresponding references.

##### *OVH*‐*based methods*

The OVH is a feature common to both atlas‐based and model‐based approaches for dose prediction, as summarized in Tables 1 and 2. Wu et al. and Kazhdan et al. introduced the concept of the OVH as a one‐dimensional function measuring the proximity of an OAR to the target.^3^, ^108^ The OVH calculation involves the uniform expansion and contraction of the target volume. Target expansion occurs until an OAR is completely overlapped by the target, and contraction is repeated until there is no overlap between the target and the OAR.^3^ The resulting OVH describes the percentage of the OAR volume that overlaps with a uniformly expanded or contracted target. In general, OVH‐driven models assume that the dose to an OAR is inversely proportional to the distance from the target. Next, we survey how the OVH‐based methods have been evolved over the years to improve dose prediction accuracy.

Several studies have combined historical data with the OVH methods to predict an entire DVH (Table 1) or one or more clinically important dose metrics from a DVH (Table 2). Wu et al. applied the OVH to head/neck IMRT planning as a quality control tool to assist treatment planners with the plan evaluation task.^3^ This initial approach was further developed in another study that used the OVH to estimate achievable DVH objectives for head/neck plans.^81^ Using an OVH model ^108^ and PCA,^46^, ^47^ Wang et al. investigated the effect of interorgan dependency and the impact of data inconsistency on dose prediction for head/neck cancer. It was suggested that interorgan dependency be incorporated in the prediction models and that data inconsistency be avoided as much as possible to improve prediction accuracy.^69^ Larger dosimetric errors were found in the head/neck region (<4 Gy for 83% of cases) as compared to the prostate region (<2 Gy for 96% cases) presumably due to interorgan dependency.^69^ Moore et al. also used OVH information to predict OAR dose metrics for head/neck and prostate IMRT plans.^109^ Yuan et al. investigated the OVH to quantify the effects of various anatomical features on interpatient OAR dose sparing using IMRT and found that several important factors are attributable to OAR sparing: the mean distance between an OAR and PTV, overlap volume between an OAR and PTV, volume of an OAR outside the field, and the geometric relationship between multiple OARs.^47^ When multiple OARs are nearby a target, separate OAR‐specific prediction models were found to be more accurate at predicting voxel doses compared to using a single training model.^110^

The OVH has been investigated under a wide variety of clinical disease sites with highly variable PTV and OARs geometries. The OVH‐based model ^81^ has also been studied for pancreatic cancer in which the OARs are larger than the PTV, part of OARs can engulf the PTV, and highly deformable organs can vary the beam configurations among different patients.^111^ Petit et al. showed that the OVH‐based predicted doses were achieved within 1 and 2 Gy for more than 82% and 94% of the patients, respectively.^111^ Further, the OVH was investigated in prostate cancer patients who had a synthetic hydrogel spacer injected between the rectum and the prostate. The spacer displaces the rectum from the treatment field for preferential sparing, and correspondingly there is a global shift of the OVH. The OVH was found to be a better feature than the hydrogel volume for predicting rectal sparing.^85^ Wang et al. used OVH to build a treatment planning QA model from consistently planned pareto‐optimal plans for prostate cancer, improving planning standardization, and preventing validation with possibly suboptimal benchmark plans.^112^

In earlier OVH‐based IMRT studies, large dose variations were reported at a given OVH distance for a specific fractional volume of an OAR.^47^, ^113^ To address the variability of the distance‐to‐dose prediction, Wall et al. studied inherent inter‐planner variations across prior plans and characterized second‐order dosimetric and anatomical factors. Out of all these factors, in‐field bladder and rectal volume showed the strongest correlation (R = 0.86 and R = 0.76) with doses. Therefore, in‐field OAR volume was incorporated into the OVH only metric.^91^ The generic OVH introduced by Kazhdan et al. relies on a DVH rather than a spatial dose distribution.^108^ McIntosh and Purdie demonstrated that incorporating spatial information into the model can improve the dose prediction accuracy in comparison to the generic OVH method. The spatial information was found to improve dose prediction accuracy for certain disease sites—whole breast, rectum, and prostate cancer—yet was less important for other sites such as the breast cavity and lung.^101^

##### *Projection*‐*based methods*

Projection‐based algorithms typically use the perspective of the beam's eye view (BEV) and statistical analysis to assess the similarity between a new case and prior cases. A best‐matched prior case may be identified based on the sum of mutual information values calculated from each BEV perspective across all of the beams in a plan. This method has been used for prostate^92^ and head/neck cancer.^97^ Good et al. calculated mutual information to determine the best match for a query case with an additional deformation step to account for any differences in PTV size and shape. More specifically, the PTV projections from a matched case were deformed to the query case's PTV projections at each BEV. This KBP approach resulted in new treatment plans with improved OAR sparing, target dose conformity, and dose homogeneity compared to the original plans.^93^

##### *DTH*‐*based methods*

The distance‐to‐target histogram (DTH) plots the fractional volume of an OAR within a certain distance of the PTV surface. In addition to volumes of the PTV and OARs, the DTH metric is also used as an input feature in ML approaches to KBP. ML techniques include multivariable nonlinear regression (MVNLR) and support vector regression (SVR).^46^ It is important to note that DTH is equivalent to OVH ^108^ when distance is Euclidean. The DTH metric was extended to a generalized distance‐to‐target histogram (gDTH) by Zheng et al. in order to account for the relative shape and spatial arrangement of multiple PTVs in head/neck cancer.^54^ In comparison to the conventional model, the gDTH model improved DVH prediction accuracy for the brainstem, cord, larynx, mandible, parotid, oral cavity, and pharynx.^54^ While this gDTH model selects similar plans with respect to an individual OAR, the concept of gDTH was further extended to develop a knowledge‐based tradeoff hyperplane model. The expanded model selects similar plans with respect to all OARs by employing a case similarity metric that is a weighted sum of gDTH Euclidean distances between two cases across all OARs.^114^ Finally, the DTH has also been utilized with multivariate regression‐based models, which is commercially available as RapidPlan^TM^ in Eclipse^®^ treatment planning software.

#### 3.1.3 Influence of various parameters

##### *Outliers*/*Data inconsistency*

Outlier detection is an important consideration when building a data‐driven dose prediction model that is generalizable to a variety of new cases. Outliers degrade the fit between geometry and dosimetry, which, in turn, can compromise model performance.^109^ Outliers may be geometric or dosimetric. Geometric outliers include large anatomical variations, such as OAR distance to the PTV. As an example, a model designed for prostate only RT should be built from prior cases only treating the prostate. The inclusion of a case treating both the prostate and pelvic nodes would be a geometric outlier. Several studies investigated the influence of outliers on model performance as shown in Table 4. Dosimetric outliers represent the presence of plans in which OARs are not optimally spared due to the planning technique. In other words, dosimetric outliers are the plans for which the re‐planning can significantly reduce OAR dose without compromising target coverage. Appenzoller et al. described a model to identify outliers from suboptimal plans and showed that excluding outliers in a refined model resulted in a strong correlation between the predicted and achieved doses after re‐planning (r = 0.92 for rectum, r = 0.88 for bladder, and r = 0.84 for parotid glands). In contrast to the negative impact of outliers on the performance of a KBP model, a previous study has shown a better OAR sparing in the presence of a small number of outliers in the KBP model.^70^ Results of a previous study showed that adding 5 to 10 outliers marginally improved salivary gland and swallowing‐muscle sparing for head/neck RapidPlan^TM^‐based KBP; however, adding more than 20 outliers showed a moderate degradation of plan quality.^70^

**TABLE 4:** A list of articles with investigations on effects of outliers on plan quality and summary of evaluation metrics used by RapidPlan^TM^ with a threshold in parentheses

Ref.	Method	Outlier
109	Restricted sum of residual (RSR)	Dosimetric
70	Regression and residual analysis	Dosimetric
115	Leverage and studentized residual	Dosimetric, Geometric
116	Regression analysis scatter plots, cook's distance	Dosimetric, Geometric
53	Model‐based case filtering	Dosimetric, Geometric
RapidPlan^TM^	Cook's distance (>10) Studentized residual (>3) Modified Z‐score (>3.5) Areal Difference of Estimate (>3)	Dosimetric and Geometric

Outlier detection has been studied for different disease sites and commercial software has metrics for identifying outliers. For pelvic RT, Sheng et al. assessed the effectiveness of outlier identification by studying the impact of both geometric and dosimetric outliers. This study suggested a greater impact of dosimetric outliers with a negative impact on both bladder and rectum model compared to geometric outliers with negative impact only on bladder model.^115^ Wang et al. studied the effects of data inconsistency with respect to planning prioritizations through a) mixed training dataset with a consistent validation dataset b) a consistent training dataset with a mixed validation dataset c) both a mixed training and validation dataset d) both consistent training and validation dataset and found that data inconsistency led to a large increase in prediction error with error_d_ < error_c_ < error_a_ < error_b_.^69^ While outlying suboptimal plans may be removed from the training cohort,^4^ an alternative that maintains the training set size is to re‐plan those cases. This approach has been tested for prostate and head/neck cancer^116^ and lung cancer.^36^ Clinically available RapidPlan^TM^ provides different statistical evaluation metrics for identifying the outliers as shown in Table 4.

##### Diversities within traditional KBP methods

In KBP, the knowledge from prior cases can be used in different combinations (e.g., creating VMAT KBP model by prior knowledge of IMRT plans for a given treatment site). Here, we present a review of studies that investigated the applicability of traditional KBP methods with respect to variations in external parameters and their combinations (e.g., cross‐modality, multi‐institution, sample size). Studies of this nature were performed on both platforms: retrospectively on a commercial KBP module (e.g., RapidPlan^TM^ in Eclipse^®^) and in‐house built KBP models. Wu et al. 2013 used the DVH objectives derived from previous IMRT plans as an optimization parameter for VMAT treatment planning in head/neck cancer, resulting in a similar dosimetric quality compared to IMRT plans.^84^ Wu et al. demonstrated that the supine VMAT model for rectal plans can optimize IMRT plans of prone patients, yielding superior OAR sparing and quality consistency than conventional treatment planning method.^117^ The prediction models trained on Helical Tomotherapy for prostate cancer were utilized to predict constraints for optimizing new RapidArc^TM^ plans. The result was similar/increased bladder and rectum doses compared to the expert plan. Delaney et al. demonstrated that using a model‐based only on photon beam characteristics could make the DVH predictions for proton therapy. This predictor could be used as a patient selection tool for proton therapy.^42^ McIntosh et al. studied contextual atlas random forest (cARF) algorithm with and without OAR region of interest features and found that the algorithm can pick better atlases without ROI features. However, the dose distribution could not be accurately mapped from those atlases onto a new patient.^102^

KBP models have successfully navigated some plan differences but not others. Huang et al. demonstrated that the RapidPlan^TM^ model for one energy (10 MV) can generate dose–volume objectives for plans with 6 and 10 MV photon beam energy; however, a RapidPlan^TM^ model for flattened beams cannot optimize un‐flattened beams prior to adjusting the target objectives.^40^ A RapidPlan^TM^ module can generate high‐quality treatment plans compared to manually optimized plans for prostate cancer.^71^ For esophageal cancers, the RapidPlan created from plans optimized using RayStation produced comparable lung doses.^38^

KBP models have been investigated for standardizing plan quality across institutions. For patients enrolled in Radiation Therapy Oncology Group (RTOG) 0617, Kavanaugh et al. showed the feasibility of a single‐institution RapidPlan^TM^ model as a quality control tool for multi‐institutional clinical trials to improve overall plan quality and provide decision support to determine the need for clinical trade‐offs between target coverage and OAR sparing.^36^ For prostate cancer, Schubert et al. have demonstrated the possibility of sharing models among different institutes in a cooperative framework.^63^ For prostate cancer RapidPlan^TM^ among five different institutions, Ueda et al. suggested that it is critical to ensure similarity of the registered DVH curves in the models to the institution's plan design before sharing the models. Good et al. applied the prostate model trained with a dataset from their institute to generate plans for patient datasets outside of their institution with the potential of homogenizing plan quality by transferring planning expertise from more to less experienced institutions.^93^ Good et al. achieved superior or equivalent to the original plan in 95% of 55 test patients.^93^ More recently, a disease site‐specific multi‐institutional, NRG‐HN001 clinical trial‐based RapidPlan^TM^ model was built as an offline quality assurance tool for which it improved the sparing of OARs in a large number of reoptimized plans submitted to the NRG‐HN001 clinical trial.^35^

##### Sample size

**FIGURE 3:**
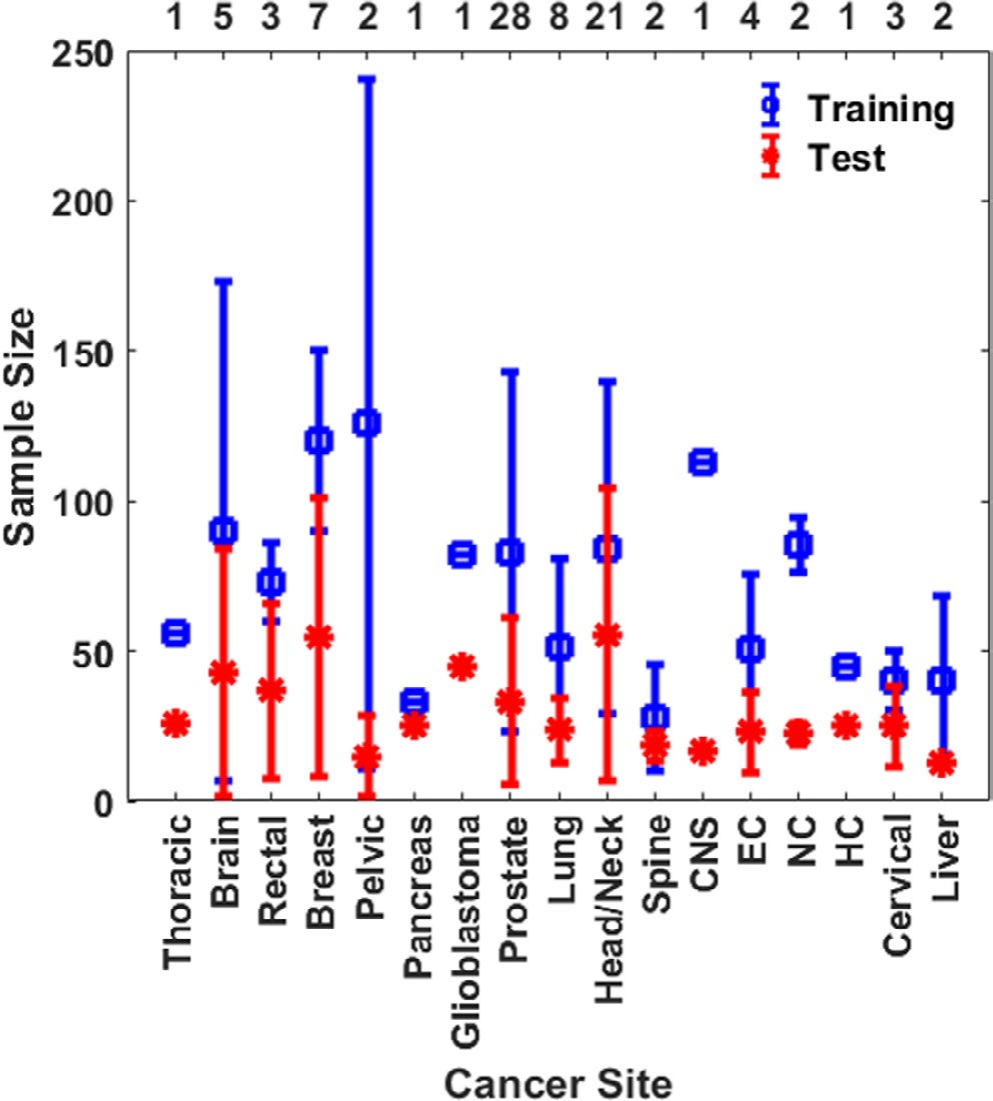
The average number of training and testing datasets in traditional KBP dose prediction methods for each cancer site. The values are averaged over the number of investigations listed on the top x‐axis and the error bars represent standard deviation. CNS, Central Nervous System; NC, Nasopharyngeal Cancer; EC, Esophageal Cancer

Figure 3 shows an average number of training and test set for each cancer site in traditional KBP methods with a standard deviation over the number of investigations listed on the top x‐axis. The number of training/test sample size was not directly mentioned or required in the methods described in some publications. We have also tabulated the list of retrospective studies performed by RapidPlan^TM^ along with their treatment sites, training, and validation dataset size in Table 5.

**TABLE 5:** Summary of published works on KBP using RapidPlan module by Varian treatment planning system

Ref.	Training size	Validation size	Cancer site	Purpose
34	57	23	Lung (VMAT)	To develop an RP‐KBP model for malignant pleural mesothelioma for patients with two intact lungs
35	50	50	Head/Neck (IMRT)	To establish a threshold of improvements of treatment plans submitted to the clinical trials for head‐neck cancer (NRG‐HN001) through a multi‐institutional KBP model
36	104	25	NSCLC (VMAT)	To evaluate the feasibility of single institution KBP model as a dosimetric quality control for multi‐institutional clinical trials to RTOG 0617
37	30, 60	13	Liver (IMRT)	To study prediction capability of RP general model (Model G with 60 cases) versus RP‐specific model (Model S with 30 cases) and benchmark against clinical plans for liver IMRT
38	40	24	Esophageal cancer (VMAT)	To evaluate RP‐KBP for training models with plans optimized with a different treatment planning system (Eclipse and RayStation)
39	48	25	Prostate (VMAT)	To demonstrate the effectiveness of RP‐KBP for hypofractionated, multi‐target prostate patients
42	30	10	Head/Neck (VMAT, Proton)	To investigate whether RP based only on photon beam characteristics can be used to generate DVH‐predictions for proton therapy and whether this could correctly identify patients for proton therapy
55	35 (LR) 30 (HR)	10 HR and 10 LR VMAT	Prostate (VMAT)	To use KBP models created from helical tomotherapy plans [35 low‐risk (LR) and 30 high‐risk (HR)] for generating plans with different techniques (VMAT)
56	79	20	NPC (IMRT)	To investigate the improvements in planning efficiency and quality for patients with NPC IMRT treatments
57	82	45	GBM (VMAT)	To create an initial RP‐based KBP model for glioblastoma (GBM) and evaluate the planning efficiency of RP‐based planning against typical manual planning
59	70	24	Esophageal (VMAT)	To evaluate the performance of the RP module for esophageal cancer VMAT
60	45	25	Liver (VMAT)	To evaluate the performance of RP‐based optimized plan against manually created plans for hepatocellular cancer for clinical acceptability
61	38	10	Spine (SBRT)	To determine if RP is effective in improving the quality and efficiency of spine SBRT planning and evaluate the model for outliers
62	40 (P) 37 (C)	10 (P) 10 (C)	Prostate (IMRT) Cervical (VMAT))	To determine whether the RP module can efficiently produce IMRT and VMAT plans in the pelvic region in a single optimization and benchmark
63	43	60 (10, 7, 6, 7,13, 10, 7)	Prostate (VMAT)	To perform the multicentric validation of RP models on seven different centers and compared with corresponding manually optimized plans
65	30,30 60	15, 15	Head/Neck (VMAT)	To study whether differences in the composition of plan libraries influenced RP results for two patient groups using three different libraries and benchmark the model versus clinical plans. To evaluate the influence of model size
64	90	20	Head/Neck (VMAT)	To evaluate the potential of RP to automate the process for identifying the quality of patient‐specific plans through the correlation between predicted and achieved mean doses to the different OAR structures
66	20, 53, 60, 100, 123	>20	Prostate (VMAT)	To evaluate the performance of RP‐KBP at multiple radiation therapy departments and check its suitability for sharing the models.
67	80	70	Rectal (SIB)	To investigate the performance of RP‐KBP compared to manually optimized clinical plans for rectal SIB cases.
68	40	11 (Int.) 22 (Ext.)	Spine SBRT	To investigate whether a validated KBP model for NRG Oncology RTOG 0631 could be used as a retrospective clinical trial quality control tool
70	70	10	Head/Neck (VMAT)	To study the influence of outliers (Suboptimal plans) on the prediction of RP plans by adding suboptimal plans into a clean model with the increment of five plans.
74	83	20	Head/Neck (VMAT)	To assess the stability of RP generated plans for a different beam geometry, different management of bilateral structures, and dose fractionations. Two models were generated: a model separating ipsi‐and‐contralateral parotids and a model associating two parotids to a single structure.
75	51	30	Prostate (VMAT)	To investigate whether RP plans created through a single optimization (without any planner intervention during optimization) are clinically acceptable for prostate cancer patients
76	51	35	Cervical (IMRT)	To demonstrate an efficient method to train, refine (i.e., according to clinical trial dosimetric objectives), and validate the KBP model for an automated quality control system
71	60	20	Prostate (IMRT)	To investigate the role KBP can play in aiding a clinic's transition to a new treatment planning system
78	81	30	Pelvic (VMAT)	TO test if RP DVH estimation can be improved interactively through a closed‐loop evaluation process
117	81	10	Rectal (VMAT)	To study whether RapidPlan model trained on a technique (VMAT) and orientation can be used for another (30 IMRT plans)
72	27, 27	25, 25	Lung (VMAT) Prostate (VMAT)	To evaluate the performance of a model‐based optimization process for prostate and lung VMAT plans and evaluate its predictive power compared to manually created plans.
73	150	70	Breast (VMAT)	To evaluate the performance of a model‐based optimization process for whole breast VMAT

Abbreviations: DVH, dose–volume histogram; IMRT, intensity‐modulated radiation therapy; KBP, knowledge‐based planning; NPC, nasopharyngeal carcinoma; NSCLC, non‐small cell lung caner; RP, rapid‐plan; VMAT, volumetric‐modulated arc therapy.

The size of the training set is an important consideration when building a KBP model. For RapidPlan^TM^, it is indicated that the minimum number of plans required for model creation is 20; however, adding additional plans will usually help create a more robust plan.^118^ Numerous studies have compared the quality of plans generated by RapidPlan^TM^ by high‐quality plans in training and found that 25–30 plans may produce a clinically acceptable plan for prostate^72^ and head/neck^65^ cancer. Zhang et al. showed that approximately 30 plans were sufficient to predict dose–volume levels with less than 3% relative error in both head and neck and whole pelvis/prostate.^79^ For prostate cancer, Boutilier et al. analyzed the effects of the training set size on the accuracy of four models from three different classes: DVH point prediction, DVH curve prediction, and objective function weights. The authors concluded that the minimum required sample size depends on the specific model and endpoint to be predicted.^119^

The requirement of sample size also partially depends on the robustness of the model used. Yuan et al. used 64 and 82 cases for prostate and head/neck case, respectively, in support vector regression (SVR) model for DVH predictions.^47^ Landers et al. demonstrated statistical voxel dose learning (SVDL) to be more robust to patient variability compared to spectral regression and SVR for noncoplanar IMRT and VMAT for head/neck, lung, and prostate cancer by 20 cases for each site in fourfold cross‐validation.^110^ An atlas‐based dose prediction^101^ is the more sophisticated method in which each patient in the training set represents 1 atlas. Feature extraction and characterization are typically performed on CT of the patients, which results in probabilistic dose estimates to find the most likely voxel dose from similar atlases. In comparison to artificial neural network (ANN) and SVR methods, large training sample sizes were required for this method (58 for rectal, 77 for lung, 97 for breast cavity, 113 for central nervous system (CNS) brain, 144 for breast, and 144 for prostate cancer).

##### Treatment planning efficiency

KBP methods may reduce the treatment planning time by minimizing the number of trial‐and‐error steps performed by a person in the treatment planning process. For the commercial KBP module, the total time including data collection, training time, generation of constraints and objectives, and optimization time can depend on the treatment type (i.e., VMAT or IMRT), design (i.e., number of arcs or angles), site (i.e., simple, or complex), and available GPU. For glioblastoma disease site, structure selection and generation of constraints and objectives took 2 minutes for both an IMRT and a 2‐arc VMAT plan, whereas the optimization and dose calculation step took 5 minutes for an IMRT plan and 11 minutes for a 2‐arc VMAT plan. Moreover, it took a planner about 4 hours to create a plan without any KBP module assistance.^57^ For nasopharyngeal cancer, Change et al. demonstrated that using RapidPlan^TM^ module reduced the average planning time substantially—(295 minutes vs. 64 minutes)—compared to the average planning time of plans created without using RapidPlan^TM^ module. For malignant pleural mesothelioma, the time taken with RapidPlan was 20 minutes compared to 4 hours for manually created plans by an experienced treatment planner.^34^ The time taken to generate objectives and optimize a plan is reported to be ranging from 15 to 120 s and 300 to 1620 s, respectively.^42^, ^55^, ^56^, ^57^, ^72^, ^73^, ^74^ The planning time when used auto‐planning by pinnacle (APP) has also been reported to be 135 min for a single arc VMAT plan for esophageal cancer.^120^ The protocol‐based APP‐based plans have been shown to reduce the manual time spend per treatment plan due to their large clinical acceptance rate.^120^, ^121^, ^122^, ^123^, ^124^ In general, the use of KBP resulted in improved treatment planning efficiency when compared with manually created plans.

#### 3.1.4 Summary

Overall, the review of traditional KBP dose prediction publications thus far suggests an improved efficiency compared to manual optimization, sufficient flexibility of traditional KBP methods in terms of their applicability (i.e., multimodality in EBRT), the need of these models for dynamic sites (i.e., pancreas), the requirement of an automated approach for accounting for outliers to further enhance the treatment planning efficiency,^70^ and the potential of building site‐specific universal RapidPlan^TM^ models for multi‐institution adaptation.

### 3.2 Deep learning

DL offers numerous advantages across the different multidisciplinary steps of radiotherapy treatment planning. In contrast to traditional KBP methods, DL methods can learn features directly from the raw dataset. Because DL methods are good at discovering intricate structures in high‐dimensional data, DL methods are able to solve a wide range of scientific problems.^125^ Neural networks underpin DL methods in learning various tasks. A multilayer perceptron has fully connected networks in which each neuron in one layer is connected to all the neurons in the next layer. Multilayer perceptron is now succeeded by CNN, a class of DNN with regularized multilayer perceptron.^126^ A CNN is by far the most widely used DNN for the dose prediction task as can be seen in Table 6. The main components of a typical CNN are convolutional layers, max‐pooling layers, batch normalization, dropout layers, a sigmoid or softmax layer. These components of CNN will be summarized below. Additionally, other neural network designs will also be described briefly for conceptual understanding prior to reviewing works on the dose prediction task.

**TABLE 6:** A list of publications on DL‐based dose prediction for various treatment sites

Ref.	Architecture	Input	Output
127	ANN	Number of fields, PTV volume, PTV to OAR distance, azimuthal and elevation angles	3D
128	ANN	Distance to PTV, Distance to OARs PTV volume	3D
129	ANN	16 different geometrical features	3D
27	Modified U‐net	PTV + OAR + Prescription	2D
25	ResNet‐50	CT + OAR + PTV images + dose distribution image	2D
26	3D‐FCN	3D CT + OAR + Prescription	3D
130	U‐Res‐Net	3D CT + OAR	3D
131	GAN	Contoured CT images + dose distribution	2D
132	HD U‐Net	OAR + PTV + Beam information with approximated dose	3D
133	CNN ‐ Res‐Net 101	Contoured images + coarse dose map, with out of field labels	2D
134	U‐Net	PET and CT image patches	3D
135	HD U‐Net	OAR + PTV	3D
136	U – Net	CT only, CT + ISO, CT + Contours, CT + ISO + Contours	2D
137	Modified 3D U‐Net	DVHs + Contours	Pareto Dose Distributions
138	U‐Net	PTV + Body + OAR, PTV + Body + OAR + Dose information from selected beam angles	Pareto Dose Distributions
139	3D U‐Net DRN	CT + FMCV	3D
140	Modified U‐Net	Density map + 3D CT +Activity map	2D
141	U‐Net	PTV + OAR contours	3D
142	GAN	CT + RT Doses, PTV + OAR	2D
143	ResNet−50	CT + OAR +PTV + body contours	3D
144	U‐Net	Low‐resolution dose + CT	2D
145	HD U‐Net	CT + RT dose distribution	3D
146	GAN	CT + PTV + OAR	2D
24	Attention gated GAN	CT + PTV + OAR	3D
147	GAN	PTV + OAR + Body	Pareto Dose Distribution
148	3D GAN	Contoured CT images	3D
149	3D U‐Net + Residual Network	CT + OAR + PTV contours + Beam + Dose	3D
150	3D U‐Net	OAR + PTV contours	2D
151	Dense‐Res hybrid Network	Beam + structural information	Static field fluence prediction
152	Virtual Treatment Planner Network	DVH	TPPs adjustment action

Abbreviations: DRL, deep reinforcement learning; DVH, dose–volume histograms; FMCV, fluence map converted volume; GAN, generative adversarial network; HD, hierarchically dense; OAR, organ at risk; PTV, planning target volume; TPP, treatment planning parameter.

The convolutional layer consists of a set of convolutional kernels where each kernel acts as a filter. First, the image receptive fields are processed through a series of convolutional kernels that aid in extracting features. Kernels use a specific set of weights to convolve with corresponding elements of the receptive field. The weight sharing ability of convolutional operation allows the extraction of different sets of features within an image by sliding kernel with the same set of weights on the image. This makes CNN more efficient than the fully connected networks. This operation can be grouped based on the type and size of filters, direction of convolution, and type of padding.^125^

From the result of the convolution operation, the feature motifs can occur at different locations in the image. The goal is to preserve its approximate position relative to others rather than the exact location. The pooling or down‐sampling sums up similar information in the neighborhood of the receptive fields and outputs the dominant response within this local region, helping to extract the combination of features that are invariant to translational shifts.^153^ Commonly reported pooling formulations used in CNN are max, average, L2, spatial pyramid pooling, and overlapping.^154^, ^155^

A nonlinear operation, also known as an activation function, helps in the learning of sophisticated patterns by serving as a decision function. Different activation functions reported in the literature are sigmoid, tanh, SWISH, ReLU,^125^ and its variants including leaky‐ReLU, parametric ReLU (PReLU) have also been used to inculcate the non‐linear combination of features.^155^, ^156^, ^157^, ^158^, ^159^ MISH is a more recently proposed activation function, which has shown better performance than ReLU on benchmark datasets.^160^ ReLU and its variants are generally preferred as activation functions because of their ability to overcome the vanishing gradient problem.^161^

Batch normalization is applied to address the issue of internal covariance shifts, a change in the distribution of hidden unit values within the feature maps that can reduce the convergence speed. Batch normalization essentially unifies the distribution of feature map values by setting them to zero mean and unit variance, which, in turn, improves the generalization of the network by smoothening the flow of the gradient.^162^

Finally, overfitting occurs when the model is trained to closely or even exactly fit a set of training data at the expense of degraded model generality as it would fail to learn general underlying patterns within the data.^163^ Different approaches such as dropouts, data augmentation, and weight regularization have been used for preventing model overfitting. While a typical neural network has all nodes activated during training, a dropout layer omits a combination of certain nodes along with their connections from the neural network each time the gradient is updated, which, in turn, prevents the network from over‐adaptation.^164^ Other forms of regularization include early stopping criterion, weight regularization, bias adding, data augmentation, and model combination. Briefly, early stopping criterion is the stoppage of the training when a specific performance measure in the form of validation loss or accuracy is reached. L1 and L2 regularization are common examples of weight regularization in which a regularization term, alpha, is added to the loss function. The idea behind the weight regularization is to find the right balance between alpha and the model complexity as it can lead to either underfitting (fails to perform well on both training and unseen dataset) or overfitting (performs extremely well on training but fails to perform well on an unseen hold‐out dataset). Data augmentation method inflates the training dataset size by warping, which preserves their labels, or by oversampling, which creates synthetic instances to increase the training dataset size.^165^

Loss function, in its simplest form, is the difference between the predicted and the target output. A loss function is presented in the form of an objective function that is to be minimized during the backpropagation step to improve the network performance. Janocha et al. investigated how different choices of loss functions affect deep models and their learning abilities, as well as robustness to various effects.^166^ While log loss performed well, other losses may also be preferable depending on the application of a given model.^166^

Backpropagation is a widely used technique in both traditional ML and recently emerging DL methods. Briefly, it systematically allows improvements in weights and biases to further improve the network's prediction in three steps: (1) feed‐forward network with learnable parameters; (2) network's performance is measured with a loss function; (3) the error is backpropagated through the network to alter the learnable parameters. This process is typically achieved through a gradient descent which allows for instantaneous rates of change between the parameter to the neural network's error. Deeper networks with many layers tend to train slowly due to gradient exploding/vanishing. For faster and efficient learning, He et al. proposed to utilize the residual functions instead of directly fitting a desired underlying mapping.^167^ A densely connected neural network (DenseNet) by Huang et al. connects each layer to every other layer.^168^ More recently, the attention gate was introduced in CNN in order to suppress irrelevant features and highlight salient features useful for a given task.^169^

#### 3.2.1 Deep artificial neural network

The simplest form of ANN consists of three layers: an input layer, the hidden layer, and the output layer. Neurons within each layer are nodes which are connected to subsequent nodes via links that correspond to biological axon‐synapse‐dendrite connections, analogous to the neural cell of human. The hidden layer is embedded between an initial input layer and the final output layer. The use of DL in dose prediction was initially utilized in the form of ANN.^127^ Therefore, ANN‐based studies are included in the DL‐based dose prediction category in Table 6. In these earlier methods, organ volumes including PTV and OARs, number of fields, and distances from OARs to the PTV were used to train ANN, which was then used to correlate dose at a given voxel to several geometric and plan parameters. The earlier ANN‐based methods were, therefore, similar to the traditional KBP methods in terms of framework. Deep ANN, moreover, consists of multiple hidden layers in addition to an input and an output layer, separating itself from the original three‐layer ANNs. In general, the number of layers determines the network's depth, and the number of neurons determines its width. Deep ANNs can learn deeper features by filtering information through multiple hidden layers. Each neuron between its input and output undergoes a linear followed by a non‐linear operation. In DL‐KBP, the ability of deep ANN to teach itself, through multiple hidden layers between input and output layer, provides the flexibility of presenting raw information without having to extract different geometric features.

**FIGURE 4:**
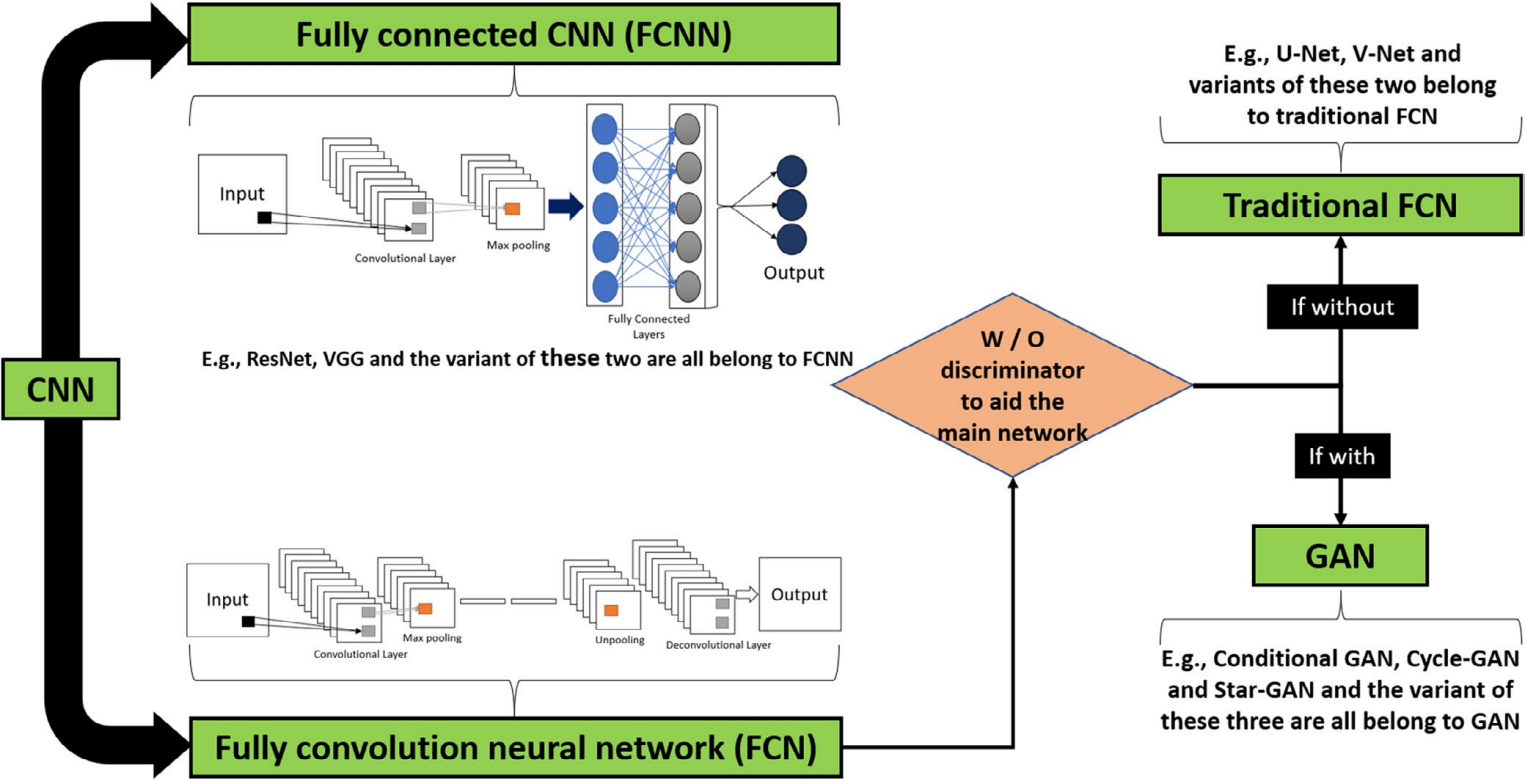
Flow chart of convolutional neural network (CNN) with its extended networks

In layered format, each neuron receives information from the neurons in the previous layer and passes processed information to neurons of the next layer. Alternatively, residual connections can be added to connect neurons in non‐adjacent layers such as ResNet proposed by He et al.^170^ The ResNet architecture has been presented with different numbers of layers: ResNet (18, 34, 50, 101, 152). Many DNN architectures have been presented for various applications. The fully convolutional neural network (FCN) and fully connected CNN (FCNN) have been applied so far for the dose prediction task (Table 6). Figure 4 shows the flowchart of the CNN and its extended networks.

#### 3.2.2 Convolutional neural network

CNNs, including fully convolutional neural network (FCN) and fully connected CNN (FCNN), have been applied so far for the dose prediction task (Table 6). Figure 4 shows the flowchart of the CNN and its extended networks. Different architectures have been proposed in the literature to enhance the performance of CNN. U‐Net, originally built for the segmentation of neuronal structures in electron microscope stacks,^23^ is one of the most widely used architectures in CNN. In addition to segmentation, it is also used for image‐to‐image translation tasks that outputs an image that has a one‐to‐one voxel correspondence with the input. U‐Net permits effective feature learning even with a small number of training sample size. Milletary et al. proposed a three‐dimensional variant of U‐Net known as V‐Net.^171^

#### 3.2.3 Generative adversarial network

Generative adversarial network (GAN) is a widely used supervised learning method in DL.^172^ Two major components of GAN are generative network and discriminator network that are trained concurrently to compete against each other. The goal of the generative network is to generate artificial data that can approximate a target data distribution from a low‐dimensional latent space, whereas the goal of the discriminator network is to recognize the data presented by the generator and flag it as either real or fake. For the dose prediction task, the artificial data generator is replaced by the predicted dose map, which then goes through the discriminator in order to be identified as a realistic or an unrealistic dose distribution. Both, dose map generator and discriminator, networks get better over the course of training to reach Nash equilibrium, which is the minimax loss of the aggregate training protocol.^172^ Some of the popular variants of GAN include CycleGAN,^173^ conditional GAN (cGAN),^174^ and StarGAN.^175^ GAN is widely used in medical imaging.^13^, ^16^, ^17^, ^176^

#### 3.2.4 Reinforcement learning

Reinforcement learning (RL) trains an agent, connected to its environment through perception and action, to make adjustments based on the interaction between the agent and the environment. The agent gets certain indications about the current knowledge of the environment at each step of its interaction. Based on this indication, the agent then chooses an action to generate an output. This action changes the state of the environment, the value of this state transition is communicated to the agent through a reward function. The agent's behavior can learn to do this over time through exploration and exploitation.^163^

#### 3.2.5 Deep learning in dose prediction

**FIGURE 5:**
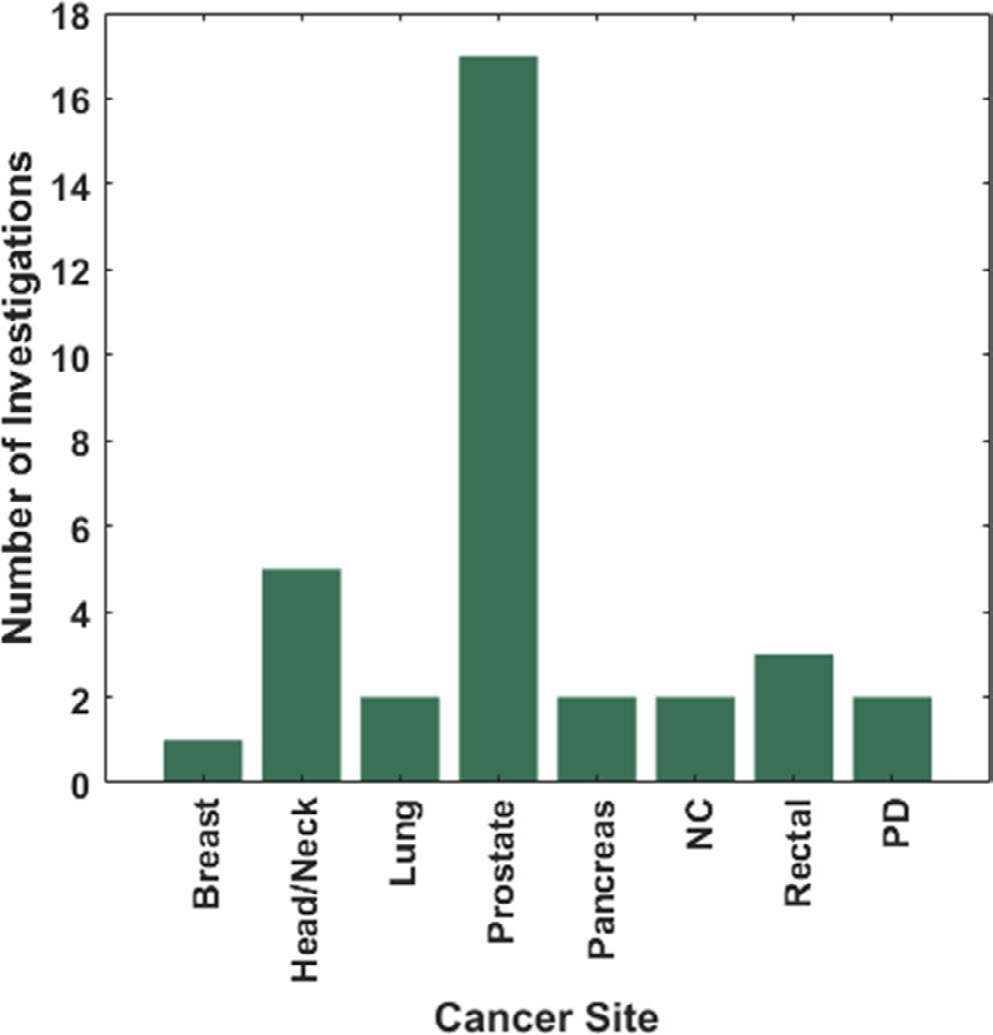
The total number of DL‐based dose prediction investigations for various cancer sites. NC, Nasopharyngeal Cancer; PD, Personalized Dosimetry

DL‐based dose prediction methods can be categorized according to DL properties such as network architectures (CNN, GAN, etc.), input image types (CT only, CT + OAR + PTV contours, etc.), output types (2D or 3D dose distribution), and sample size (training, testing, etc.). As shown in Figure 1, DL‐based dose prediction methods have gained popularity among the researchers only in the past few years, there are nearly 30 publications on DL‐based dose prediction as of August 2020. These DL‐based dose prediction publications are tabulated in Table 6 along with their network architectures, input, and output characteristics. Figure 5 represents the total number of DL‐based dose prediction investigations per treatment site. This follows a similar trend to that observed for traditional KBP methods with the highest number of investigations being on prostate and head/neck cancer sites. Here, we categorized DL‐based dose prediction publications thus far into three groups based on network architectures: I) CNN – namely U‐Net architecture, II) GAN, and III) RL. We first provide the review of work for each network architecture followed by their applicability on various dose prediction applications and limitations. Subsequently, we discuss the influence of different parameters in DL‐based dose prediction methods.

#### 3.2.6 *Overview of CNN*‐*based works*

As shown in Table 6, U‐Net has been widely used CNN architectures used for predicting dose distributions. U‐Net is effective in terms of end‐to‐end learning of global and local features because it consists of encoding and decoding paths. The decoding path concatenates the features from both previous layers in the encoding path and features from current layers in the decoding path. Many variants of U‐Net including 3D U‐Net have appeared in the literature for dose prediction purposes (Table 6).

Earlier work in DL‐based dose prediction methods involved predicting doses in a 2D manner.^25^, ^27^ Sumida et al. used the U‐net model, initially proposed by Ronneberger et al.^23^ to make 2D dose prediction. The network was trained to make dose prediction for Acuros XB (AXB) from low‐resolution dose calculated through AAA algorithm and CT.^144^ Similarly, Nguyen et al. also trained a seven‐level hierarchy with a modified version of the original U‐Net to make dose prediction for a prostate case.^27^

More recent works were focused on predicting 3D dose distributions using DL methods. To overcome increased computation load in 3D dose prediction, Nguyen et al. proposed Hierarchically Densely U‐Net (HD U‐Net), which not only was able to predict 3D dose distribution, but also outperformed dose predictions made by the standard U‐Net model.^135^ HD U‐Net combines DenseNet's efficient feature propagation and utilizes U‐Net's ability to infer both local and global features by connecting each layer to every other layer in a feed‐forward fashion, yielding better RAM usage, and better generalization of the model. To further simplify the 3D dose prediction problem and increase prediction accuracy, Xing et al. projected the 2D fluence maps onto the 3D dose distribution using a fast and inexpensive ray‐tracing dose calculation algorithm and trained HD U‐Net to map the ray‐tracing low accuracy dose distribution (does not consider scatter effect) into an accurate dose distribution calculated using collapsed cone convolution/superposition algorithm.^145^

DL‐based methods have also been expanded to predict pareto optimal dose distributions so that physicians can learn the desired dosimetric trade‐offs in real‐time and learn the viability of different dosimetric goals. Ma et al. constructed the 3D U‐Net architecture to predict individualized dose distribution for different tradeoffs.^137^ In predicting the pareto dose distribution, the network should be able to map many dose distributions from single anatomy, and in doing so differentiate between the clinical consequences of corresponding predicted dose distributions. To address this clinical tradeoff problem across different dose distributions, Nguyen et al. proposed the differentiable loss function based on the DVH and adversarial loss in addition to traditional voxel‐wise mean square error (MSE) loss to train the network.^147^ Along the same line of work, Bohara et al. incorporated beam information to predict pareto dose distribution using anatomy beam model proposed by Barragán‐Montero et al.^138^

U‐Net architecture has also been used for radiopharmaceutical dosimetry.^134^, ^140^ The network was trained to predict 3D dose rate maps given the mass density distribution and radioactivity maps. The current clinical standard is the decades‐old Medical Internal Radiation Dose Committee (MIRD) formulism which is limited by somewhat crude analytical equations. The long‐term goal of the U‐Net studies is to create a stable DL‐based dose estimation model that achieves a precision close to that of Monte Carlo simulations.

He et al. proposed the residual network, known as ResNet, to mitigate the difficulty of training DNN caused by gradient vanishing.^170^ He et al. reformulated the layers as a learning residual function instead of directly fitting a desired underlying mapping. Chen et al. and Fan et al. proposed the DL method based on ResNet with 101 and 50 weight layers, respectively, to predict dose distribution for head/neck cancer IMRT patients.^133^, ^143^ Since networks with very deep layers are difficult to train due to vanishing gradient, such networks used shortcut connections to add to the outputs of the stacked layers.^170^ More recently, Liu et al. proposed ResNet for dose prediction in the nasopharyngeal cancers for Helical Tomotherapy. To achieve multi‐scale feature learning, Liu et al. divided the ResNet into several parts without fully connected layers and respectively combined with input data to achieve pixel‐wise feature abstraction and extraction in the structural image.^130^

#### 3.2.7 *Overview of GAN*‐*based works*

GAN entails a pair of neural networks: a generator and a discriminator. From the treatment planning standpoint, the generator could be represented as the treatment planner who generates the plan, and the discriminative network could be represented by a radiation oncologist who evaluates the generated plan. Both the treatment planner and a radiation oncologist get better at performing their tasks as they become more experienced over time. Only a handful of studies have investigated the performance of GAN for dose prediction task as shown in Table 6.

Mahmood et al. demonstrated the first use of 2D GAN for predicting dose for each 2D slice independently for oropharyngeal cancer. Subsequently, Babier et al. proposed the first 3D GAN for the prediction of full 3D dose distributions, which outperformed the 2D GAN model proposed by Mahmood et al. presumably owing to its ability to learn the 3D features in contrast to 2D features by 2D GAN networks. Recently, Vasant et al. proposed a novel 3D attention‐gated generative adversarial network (DoseGAN) as a superior alternative to the current state of the art dose prediction networks.^24^ Spatial self‐attention allows networks to emphasize portions of the intermediate convolution layers. Attention gated GAN can potentially offer deeper and more efficient discrimination, while being trained in parallel with the generator network and facilitating the model convergence.^24^, ^177^ This addresses the issue of keeping the number of network parameters as low as possible in conventional GAN. Attention‐gated GAN proposed by Vasant et al. outperformed conventional 2D and 3D GAN in all dosimetric criteria including PTV and OARs.^24^

All four studies ^24^, ^131^, ^142^, ^148^ on GAN‐based dose predictions constructed a generator and discriminator network using the pix2pix architecture proposed by Iosa et al.^178^ In these studies, U‐Net is used as a dose map generator that passes a contoured CT image slice through consecutive layers, a bottleneck layer, and subsequent deconvolution layers. U‐net also uses skip connections to easily pass high‐dimensional information between the input (CT image slice or contoured structures) and the output (dose slice).

#### 3.2.8 *Overview of RL*‐*based works*

RL is a unique framework that resembles the workflow of treatment planning optimization. RL has been combined with DNN to accomplish human‐level performances for decision‐making tasks.^179^ In order to implement the RL‐based framework for dose prediction task, in‐house treatment planning system and RL architecture must be synchronized in a single pipeline, which is implemented by only a few research groups.^151^, ^180^ Recently, RL was used to train a DNN named virtual treatment planner network, which learns to adjust treatment planning parameters to improve plan quality similar to the treatment planning process.^152^, ^180^ The use of the trained network to perform treatment planning improved plan quality score compared to the initial plan. In comparison to U‐Net and GAN, deep RL is one of the least studied networks for dose prediction tasks presumably due to the high action space of the actual treatment planning process.

#### 3.2.9 Overview of learning processes

Three commonly used learning processes include unsupervised learning (USL), semi‐supervised learning (SSL), and supervised learning (SL). In this section, we briefly present a review of SL due to its wide adoption in dose prediction application. SL refers to a technique that utilizes labeled data with input‐output correspondence to train the model. The learning goal during training is defined by the paired input data and output target. While both, traditional KBP and DL‐based KBP, methods largely use the SL framework to train models, each presents a different framework with regards to labeled data with input. For instance, one subcategory of SL is regression analysis, which establishes a relationship among the variables by estimating how one set of variables affect their corresponding response variables (i.e., relationship between the distance of OAR to target and doses to OAR). As can be seen in Tables 1, 2, 3, various regression analyses were performed in traditional KBP methods. DL‐based KBP methods use CNN to learn contour‐to‐dose mappings in a supervised manner with the clinically generated dose map being the learning target. The discriminator in the GAN‐based model learns to discriminate between predicted dose map and real clinical dose map, therefore, belongs to supervised learning. Therefore, the network is trained through adversarial loss and able to predict more realistic dose distributions.^177^

#### 3.2.10 *Influence of various parameters on DL*‐*based model performance*

##### Input parameters

In terms of the number of input parameters, Williems et al. studied the impact of four different inputs (Table 6) for dose prediction under with and without data normalization of dose distribution. The order of models in terms of performance was CT + isocenter + contours >CT + contours >CT + isocenter >CT only. While the dose distribution normalization had more benefits for CT + contours, it was found to be less necessary for CT +isocenter + contours model. Whereas, normalization produced hot and cold spots for CT + isocenter model.^136^

While many studies use only CT with anatomical information (i.e., PTV and OAR contours) as inputs to the CNN^27^, ^132^, ^135^ as can be seen in Table 6, Barragán‐Montero et al. included beam geometry information along with anatomical information as inputs. As a result, the model was able to learn from the database that was heterogeneous in terms of beam configurations (i.e., noncoplanar),^132^ which was the limitation of the network proposed in the earlier studies.^27^ For rectal cancer IMRT, Zhou et al. showed improvements in the prediction accuracy by including beam configurations as input to the network compared to that of without beam configurations.^149^ For head/neck cancer, Chen et al. investigated the influence of adding out of field labels into the network training to deal with inability of 2D network to account for radiation beam geometry. It resulted in a better overall performance compared to the network excluding out‐of‐field labels.^133^ For prostate cancer, Murakami et al. compared the performance of CT‐only‐based GAN with contour‐based GAN in predicting target dose map and found prediction performance of contoured‐based GAN to be superior.

##### Loss functions

In terms of losses, MSE is one of the most widely used cost functions in DL methods as it has many desirable properties from an optimization standpoint. Owing to its simplicity, well‐behaved gradient, and convexity, majority of previous studies including the ones shown in Table 6 utilized only MSE loss for dose prediction. Nguyen et al. combined domain‐specific loss function based on DVH with adversarial loss and MSE loss for the training of deep neural networks. While this approach outperformed dose predictions compared to the MSE‐based trained model, for the same computational system, it increased the training time to 3.8 days with 100000 iterations compared to 1.5 days for MSE only based network.^147^

Lee et al. and Chen et al. utilized mean absolute error (MAE) cost function between the ground truth and dose rate map predicted by CNN.^133^, ^134^ As compared to MSE, MAE is more robust to outliers but may be less efficient to find the solution, whereas MSE provides a more stable and closed‐form solution. Other loss functions may include Huber loss, smooth mean absolute error, quantile loss, and log cosh loss function. So far, the MSE loss function has been the standard cost function used in DL‐based dose prediction studies.

##### Sample size

**FIGURE 6:**
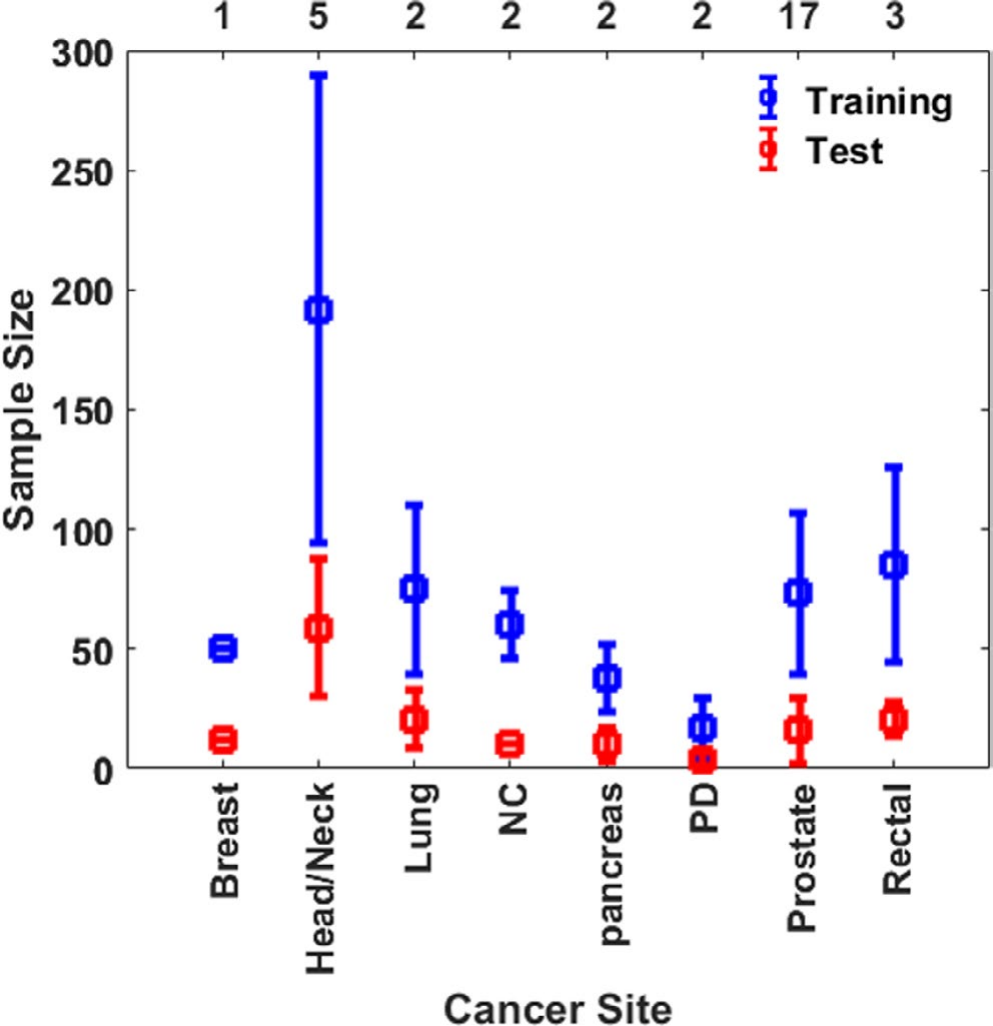
The average training and testing sample size in DL‐based dose prediction methods for each cancer site. The values are averaged over the number of investigations listed on the top x‐axis and the error bars represent standard deviation. NC, Nasopharyngeal Cancer; PD, Personalized Dosimetry

In general, the DL‐based methods require a large number of high‐quality data to be effective. Small datasets in DL can be challenging as it may result in overfitting. Data augmentation,^144^ dropout layer,^164^ estimation based on the training and the validation curves,^135^ synthesizing new data based on physics principles,^181^ and incorporating regularizations to model parameters ^182^ have been used in the literature to prevent overfitting. The process of data augmentation, more commonly used in dose prediction approaches, is to expand the dataset by synthesizing additional realistic samples from available samples. It is important to note here, however, that the process of augmentation to be used depends on the suitability of the context. For the purpose of dose prediction, we have presented the average training and testing sample size for each treatment site in Figure 6 for all DL‐based dose prediction methods to date, which provides the readers with an approximate range of training and testing dataset for each cancer site.

As shown in Table 6, three investigations on prostate cancer have been reported for predicting pareto optimal dose distributions.^137^, ^138^, ^147^ For each patient in the training set, 10, 100, and 500 plans were generated by Ma et al. Nguyen et al. and Bohara et al. respectively, to sample the pareto surface with different tradeoffs. An optimal number of plans per patient in training set is unknown as it may depend on case to case basis. Nonetheless, in the case of predicting pareto optimal plans, it may be ideal to stay within a clinically relevant regime by including only those plans that cover dosimetric trade‐offs presented by a physician.

Kandalan et al. studied the issue of generalizing DL‐based dose prediction models and make use of transfer learning to adapt a DL dose prediction model to different planning styles in the same institutions and planning practices at different institutions. A source model was adapted to four different planning styles only with 14–29 cases.^150^ A long‐term goal of these studies is to generate a universal model that can easily be transferred to different institutions for a similar task.

## 4 DISCUSSION

With the goals of improving plan quality and efficiency, researchers have investigated KBP to guide treatment planning for a new patient. In the last decade, there has been a rapid growth in the number of publications in traditional KBP dose prediction. More recently, the number of publications on DL has increased exponentially because of its flexibility and superior performances compared to many state‐of‐the‐art techniques. Over 90 articles have been published on traditional KBP dose prediction methods between 2011 and August 2020, whereas over 17 publications have already been published on DL‐based dose prediction as of August 2020.

In general, most publications demonstrate improvements in comparison to manually optimized clinical plans in terms of both treatment planning quality and efficiency. A large number of manuscripts were published on traditional methods between 2015 and 2018, with the highest number of publications in 2017. This is presumably due to the commercialization of the RapidPlan^TM^ in Eclipse^®^ treatment planning software in 2014, which allowed researchers from different centers to perform retrospective studies for investigating the influence of various parameters on the quality of plans generated through commercial KBP module. In addition to RapidPlan^TM^, RayStation allows a scripting option that allows user to implement a mathematical dose prediction model as demonstrated in.^89^ However, the automatic treatment planning module of RayStation has not been largely investigated as it may not be widely available to the users to perform retrospective investigations. An auto planning by Pinnacle (APP) is another commercially available software. It is a protocol‐based method that performs iterative optimization steps to reach a final plan compared to using the machine learning‐based method.^122^ Nonetheless, we have also tabulated these additional APP‐based clinical studies with their key findings in Table 7.

**TABLE 7:** Summary of research papers on automatic plans generated in Pinnacle (APP) with their key findings

Ref.	Cancer Site	Key Findings
183	Esophageal	APP plans were preferred for 31/32 patients and achieved lower mean doses to the lungs. APP plans and manually created plans achieved similar target coverage
124	Advanced head/neck	In a 10 patients’ study, the human‐driven plans achieved better target homogeneity, whereas the APP consistently achieved better OAR sparing. Overall, an improved tradeoff process between homogeneity and OAR sparing in APP could further enhance its benefits.
184	Oropharyngeal	The performance of cross‐institutional OVH‐based KBP compared to APP was studied on 25 patients and the plan quality was found to be comparable for both techniques.
185	Oropharyngeal	APP plans were clinically acceptable and achieved comparable PTV coverage with better OARs sparing
121	Breast	In a 25 patients’ study, dose homogeneity was found to be significantly better in APP plans compared to manual plans (*p* < 0.001).
186	Prostate	In a 23 patients’ study, doses to the PTV and rectum by APP were comparable to manually optimized plans. APP plans achieved lower doses to bladder and femoral heads compared to manual plans (*p* < 0.05)
187	Nasopharyngeal	Impact of APP was studied on IMRT and VMAT plans on 10 patients with three dose level target volumes. Better parotid sparing was achieved by APP‐VMAT versus APP‐IMRT (*p* < 0.01). PTVs coverage was comparable in both techniques.
123	Head/Neck	APP produced clinically acceptable plans in all 26 cases with better sparing of OARs. APP plans achieved a comparable plan quality score to the previously delivered plans in 94% of the evaluations.
188	Prostate	In 100 patients’ study, 98 plans met all clinical constraints with significant improvement bladder and rectum sparing in higher dose region (V72 Gy). Proposed automated treatment planning workflow reduced operator time to less than 5 min.
189	Head/Neck	Script‐based APP plans achieved higher points compared to manually created plans (67.0 vs. 62.3), however, resulted in increased monitor units up to 35.5%.
190	Whole brain	In a 10 patients’ study, APP‐based 2 coplanar VMAT and 9‐field plans were compared. With comparable dosimetric results, both APP‐based plans achieved clinically acceptable and deliverable plans and eliminated the need of generating pseudo‐structures by the planners.
191	Breast (Tangential)	Automated planning was found to be applicable in 1661/1708 patients with treatment planning time to be around 5 to 6 minutes on standard commercially available planning system hardware.
192	Lung SBRT	In a 56 patients’ study, APP plans significantly reduced D_2%_ for the spinal cord, esophagus, heart, aorta, and main stem bronchus and maintained target coverage compared to manually created treatment plans.
193	Liver SBRT	In a 10 patients’ study, APP plans resulted in comparable to manual plans, but with reduced doses to the spinal cord and planning time.
194	Prostate	Evaluated the adaptability of APP across clinics with different planning protocols and demonstrated that APP configurations can be shared and implemented across multiple centers.
195	Partial breast irradiation	In a 23 patients’ study, APP plans were compared with manually created plans. No significant differences in OARs were observed, but APP plans achieved significant improvements in PTV coverage compared to manual plans.
196	Nasopharyngeal	In a 25 patients’ study, APP and manual plans had similar ratings including PTV coverage and doses to OARs for 19/25 patients. APP plans reduced planning duration time by 17%
197	Locally advanced Head/Neck	The performance of 1) APP plans 2) RapidArc‐based automatic interactive optimizer 3) RapidPlan 4) RapidPlan with automated setup fields and 5) RayStation multicriteria optimization was studied retrospectively on 16 head/neck cases. All systems generated comparable treatment plans, but APP plans performed the best for parallel organs with minor dose differences.

Abbreviations: APP, Auto planning by Pinnacle; D_2%_, Dose received by 2% of the volume; OARs, organs at risk; PTV, planning target volume.

In terms of modality, both (traditional KBP and DL KBP) methods were mostly applied to IMRT, VMAT, and other noncoplanar intensity‐modulated external beam radiation therapy treatments. Only a small number of studies were reported for the purpose of magnetic resonance imaging‐guided therapy (MRgRT).^129^ The number of traditional KBP and DL‐based publications for on‐table adaptation may increase in the future, owing to recent technical developments such as MR‐Linear Accelerator (MR‐Linac). In terms of treatment sites, prostate, head/neck, and lung were among the most investigated sites in both traditional KBP and DL‐based methods. Other disease sites—complex abdominal or cranial—were studied less often. This trend was anticipated as both KBP techniques require large training sets, and prostate, lung, and head/neck are among the more common and static disease sites treated with external beam radiation therapy. Therefore, the availability of a prior plan is likely to be one of the predictive factors for future model development.

In KBP, three commonly reported dose prediction metrics in the literature are the entire DVH curve (Table 1), one or more dose metrics (Table 2), and voxel‐based dose prediction (Table 3). For DVH only prediction model, DTH of the OARs, volumes of PTV OARs excluding external or body structure, and patient‐specific anatomical information are used as the input to the model, whereas the output is the DVH curve of each OAR (i.e., bladder and rectum). In this case, dose outside of the PTV region may not be accounted for unless body structure is included in the model, which, in turn, would provide information regarding dose conformality or gradient outside the PTV. However, including the body structure in the model has not been a common practice for the DVH‐only prediction model.^46^ Moreover, the voxel‐based dose prediction model predicts the dose distribution accounting for doses to each voxel within the patient's image set,^96^, ^127^ hence provides spatial information including dose gradient outside of the PTV and dose conformality. However, the voxel‐based approach relies heavily on the quality of the plans used to build the model as the inclusion of outliers can compromise the model performance. Even for RapidPlan^TM^‐based KBP, several studies indicated the need to investigate the proper identification of outlier plans.^60^, ^65^, ^72^ Outlier identification in RapidPlan^TM^ involves statistics and regression plots for each structure, suggesting Cook's Distance >10.0, Studentized Residual >3.0, Areal Difference of Estimate >3, and Modified Z‐score >3.5 as potential outliers.^118^ To an extent, this also requires removal of outliers in an iterative manner with either stopping the removal once no significant improvement is observed or identification of the outliers followed by the re‐planning of all the outliers so that it can be reused in the training cohort.^116^ The time required to address the issue of outliers may vary across institutions, as those without standardized contouring and planning techniques may have many dosimetric outliers, which in turn can result in a time‐consuming process of eliminating outliers either through visual inspection or additional statistical analysis. In the literature, there only limited effort toward establishing a systematic process for identifying dosimetric and geometric outliers. To our knowledge, currently, there is no well‐established workflow for outlier identification and mitigation in terms of model creation for both KBP techniques. Therefore, a standardized automated method of outlier identification and model creation could further enhance the treatment planning experience.^53^

KBP research is growing rapidly. Past work has been surveyed before, though much has changed since then and even old work can be given a new context in light of more recent advancements. In contrast to a previous review that calculated an average number of training and testing dataset used in each year,^28^ we calculated the average number of training and testing datasets used for each cancer site for traditional KBP and DL KBP in Figures 3 and 6, respectively. Table 5 provides site‐specific training and validation dataset size for the RapidPlan^TM^ KBP module. This could inform readers of a range of training sample size used in the literature for each treatment site. Direct comparison of training sample size between the traditional and DL‐based KBP was not made as DL‐based dose prediction is a relatively new technique with a fewer number of investigations per site compared to traditional KBP methods.

There are key differences between traditional KBP and DL methods for dose prediction. In contrast to DL, an inherent limitation of traditional methods is that it is unable to extract important features and patterns hidden within the raw dataset. Both, similarity measures in atlas‐based methods and input features to model‐based methods require considerable effort to design handcrafted features (i.e., overlap volume histogram, OAR distance to the PTV, projections, etc.) that can be processed either to identify the best‐matched case or into a representation from which patterns within the input can be classified through a classifier. In traditional KBP, PCA has been widely used in the literature for feature selection owing to its simplicity. However, a major limitation of PCA is that it learns a low dimensional representation of data only with a linear projection. Whereas DNNs can be used to address this issue and untangle non‐linear projections. For instance, an autoencoder is a type of neural network that is consisted of encoder, which encodes the input into a low dimensional latent space, and decoder, which restores the original input from the low dimensional latent space.^198^ DL‐based dose prediction methods use an autoencoder type of neural network as shown in Table 6.

Multiple DL networks have been investigated for dose prediction. The two most widely investigated networks, thus far, included CNN and GAN. From the results so far, it appeared that GANs may be a good choice for dose prediction tasks over conventional CNNs for several reasons. First, GAN has been proven to perform well in lesion detection and data augmentation tasks.^148^, ^199^ In addition, GAN does not rely on pure spatial loss, such as mean square error between dose–volumes, which makes it a suitable candidate not only for the dose prediction of conventional radiation therapy but also for SBRT in which dose heterogeneity is prevalent.^177^ Furthermore, Babier et al. found that GAN models did not require significant parameter tuning and architecture modifications during implementations compared to other conventional methods.^148^ However, in contrast to CNN, one limitation of conventional GAN is that they are difficult to train and require the number of network parameters to be as low as possible. Future studies are anticipated to account for such shortcomings by proposing the extension of networks such as attention‐gated GAN.^24^ Finally, Deep RL is one of the least studied networks for generating a treatment plan presumably due to the high action space of the actual treatment planning process. One application of RL in dose prediction would be to adjust treatment planning parameters such as dose–volume objectives, constraints, and corresponding weights to reach the desired treatment plan. However, this would require synchronizing DL framework and inverse optimizer in a single pipeline, which may not be feasible with inverse optimizer of commercial TPS due to its “black box” nature. Therefore, applying RL for generating a treatment plan may require in‐house built TPS ^152^ that can be presented with DL network architectures in the same pipeline.

### 4.1 Method comparison of KBP dose predictions

KBP model performance varies as does the geometric and dosimetric complexity of treatment plans for different disease sites. For these reasons, KBP performance is presented here for specific disease sites. For head/neck cancer, the difference between the traditional KBP predicted and actual median doses for the parotids ranged from −17.7% to 15.3%,^48^ whereas it ranged between – 7.7 and 13.5% for DL‐based dose prediction.^133^ With the same level of prediction accuracy, DL‐based KBP was able to predict median dose for 80% of parotids compared to 63% by the traditional KBP method.^133^ Kajikawa et al. made the direct comparison of dose distribution predicted by the DL method with that generated by RapidPlan^TM^ for prostate cancer.^141^ This dosimetric comparison showed that CNN significantly predicted DVH accurately for D_98_ in PTV‐2 and V_35_ V_50_, V_65_ in the rectum. Given that features automatically extracted by DL methods can include both geometric/anatomic features and the mutual tradeoffs between the OARs, it gives an edge to DL methods in terms of dose prediction accuracy compared to traditional KBP methods that mainly rely on DVH and geometry‐based expected dose.

For oropharyngeal cancer, Mahmood et al. directly compared the GAN approach for generating predicted dose distribution with several traditional approaches including bagging query ^3^, ^200^ and generalized PCA,^47^ random forest.^102^ Through the gamma analysis,^201^ Mahmood et al. demonstrated that GAN plans were the most similar to the clinical plans and achieved 4.0% to 7.6% improvements in the frequency of clinical criteria satisfaction compared to traditional approaches.^131^

For prostate SBRT, Vasant et al. compared the performance of the proposed attention gated GAN with an earlier approach that used relative distance map information of neighboring input structures.^127^ In contrast to conventional radiation therapy, SBRT produces hot spots within the target volume. The mean absolute difference in V_120_ between KBP‐like approach and actual plan was fourfold higher compared to that achieved by the attention gated GAN technique, demonstrating the ability of a DL‐based method to predict cold spots and hotspots that are prevalent in SBRT dose distributions. While both, traditional and DL‐based KBP methods used the data from previously treated patients to make dose prediction for a new patient, DL‐based methods have been shown to outperform several traditional KBP methods as demonstrated by some studies in the literature.^131^, ^141^

### 4.2 Future trends

According to published articles on each KBP method in recent years, a bulk of research on further developments of models has shifted from traditional KBP methods to DL‐based methods. This is presumably due to the recent success of DL in various medical applications as well as its potential in dose prediction task. In terms of traditional KBP methods, future investigations are anticipated to be retrospective in nature by clinically available tools (i.e., RapidPlan^TM^, APP). Since DL‐based methods appear to be in their initial development stage, their possibilities are expected to be further explored through the development of new DL models and in different areas of dose prediction tasks in treatment planning workflow including adaptive radiotherapy in the near future.

Adaptive radiotherapy (ART) involves adjusting dose distribution based on anatomical changes observed on intra‐procedural imaging such CBCT. The standard approach requires a physician to perform the recontouring of OAR and tumor regions followed by plan re‐optimization, which is difficult to implement with a patient on the table due to time constraints. To date, only one study has been reported to adopt DL methods for the purpose of ART of head/neck cancer.^146^ The future trend will certainly be toward utilizing DL‐based methods to present dose prediction models of dosimetry changes and radiotherapy response for ART.

Post‐dose prediction, the main component of treatment planning workflow includes ensuring the achievability of the predicted dose plans, which often involves inverse treatment planning through manual intervention. Only a handful of studies extended such KBP in a fully automated pipeline that not only predict the dose distribution but also generates a complete treatment plan with minimum human interaction in traditional ^52^, ^101^, ^184^, ^202^, ^203^ and DL‐based methods.^131^, ^148^, ^151^ The accurate deliverability of the predicted plans is crucial and it must account for various mechanical, physical, and algorithmic constraints. It is important to note here that good predictions with the low error may not necessarily lead to the final deliverable plan with the same performance on clinical criteria. For instance, five of the seven prediction methods investigated by Babier et al. resulted in significantly worse clinical criteria satisfaction despite lower error post‐dose predictions.^148^ We, therefore, believe synchronizing an inverse optimization engine with dose prediction methods hold great potential in improving treatment planning efficacy and efficiency of DL KBP. Alternatively, a DL‐based fluence prediction has also been proposed for real‐time prostate treatment planning.^151^ This approach follows the conversion of predicted fluence maps to a deliverable treatment plan through delivery parameter generation and dose calculations directly in a treatment planning software. Such approaches do not require an inverse optimization process and involve minimal human intervention. After generating a clinically acceptable plan, a subsequent task in many clinics involves patient‐specific quality assurance (QA) measurements that are performed routinely prior to actual treatment delivery. This QA step is not feasible if adapting a plan for a patient currently on the treatment table. Several ML^204^ and DL^205^ approaches have been reported for predicting gamma analysis pass rates for patient‐specific IMRT QA that may prove to be an acceptable surrogate for actual measurements or be an acceptable complement in an overarching QA program. More effort is needed to incorporate such approaches into the treatment planning pipeline to establish a fully automated workflow.

One of the challenges in data‐driven algorithms, including both ML and DL, is that it requires a large set of high‐quality data. Since the quality of data and radiotherapy practices vary from one center to the other, the heterogeneity in previously treated plans becomes a major obstacle in the deployment of data‐driven solutions in the field of radiation oncology. To address this issue, the concept of transfer learning for model adaptation to different learning styles at different centers may be investigated further in the future. A long‐term goal of this area of investigations would be to incorporate data‐driven predictive tools as a part of the clinical pathway. Pooled data from multiple institutions are the likely path forward for creating KBP models for rare disease sites.

Finally, while the emergence of traditional ML and modern DL methods in the form of KBP has had a positive impact in radiotherapy clinics, adopting several practices from computer vision (CV) communities can further contribute to the growth of KBP on a larger scale. Data sharing is one category of CV that focuses on collaborative analysis and publishing on the web.^206^, ^207^, ^208^ Within KBP communities, an exclusive platform may be used to share, combine, and analyze the dataset within the boundaries of HIPAA (Health Insurance Portability and Accountability Act). Through collaborative analysis, individuals from different centers can run KBP models on the dataset from their centers and later merge with other versions of the data available on this platform. Practice of publishing code is another area that the KBP community may adopt from CV as it matures. Availability of codes along with a corresponding paper on web (i.e., paperswithcode.com) aids the reproducibility of research, and provides useful information and details that may not be sufficient to provide in publications (i.e., training strategies, detailed list of hyper‐parameter values, network structure, data augmentation, etc.). Eglen et al. compiled general guidelines from different areas of science for sharing computer codes and programs.^209^ By advocating for such guidelines, the KBP community may embrace reproducibility, transparency, and reusability of research products.

## 5 CONCLUSION

In the last decade, a tremendous amount of work has been done to improve treatment plan quality and efficiency through knowledge‐based planning research. We have reviewed over 120 articles covering two major KBP methods for dose prediction: traditional KBP methods and the more recent DL‐based KBP. Many traditional KBP methods are shown to be equivalent or superior to an experienced planner with greater efficiency. Recent developments in DL‐based KBP methods also hold a great potential to further improve the accuracy of the dose prediction task without compromising on efficiency. Both traditional and DL‐based KBP methods need further development for many other disease sites. Given the commercial availability of the traditional KBP module, more retrospectives studies are foreseen in the future. However, new DL‐based KBP methods are actively being introduced and trending in a steep upward direction and its commercialization may be anticipated in near future. There are various areas of future research, several of which have been highlighted in this review, required to achieve an ultimate goal of a fully automated treatment planning system.

## CONFLICT OF INTEREST

None.

## AUTHOR CONTRIBUTIONS

Shadab Momin: Conceptualization, Methodology, Investigation, Writing ‐ Original Draft, Visualization Yabo Fu: Methodology, Writing ‐ Review and Editing Yang Lei: Methodology, Writing ‐ Review and Editing Justin Roper: Writing ‐ Review and Editing Yang Lei: Writing ‐ Review and Editing Jeffrey D. Bradley: Writing ‐ Review and Editing Walter J. Curran: Writing ‐ Review and Editing, Supervision Tian Liu: Writing ‐ Review and Editing, Supervision Xiaofeng Yang: Conceptualization, Writing ‐ Review and Editing, Supervision, Project administration, Funding acquisition.
